# Effect of Glut‐1 and HIF‐1α double knockout by CRISPR/CAS9 on radiosensitivity in laryngeal carcinoma via the PI3K/Akt/mTOR pathway

**DOI:** 10.1111/jcmm.17303

**Published:** 2022-04-12

**Authors:** Yang‐Yang Bao, Jiang‐Tao Zhong, Li‐Fang Shen, Li‐Bo Dai, Shui‐Hong Zhou, Jun Fan, Hong‐Tian Yao, Zhong‐Jie Lu

**Affiliations:** ^1^ Department of Otolaryngology The First Affiliated Hospital Zhejiang University School of Medicine Hangzhou City China; ^2^ State Key Laboratory for Diagnosis and Treatment of Infectious Diseases The First Affiliated Hospital Zhejiang University School of Medicine Hangzhou City China; ^3^ Department of Pathology The First Affiliated Hospital Zhejiang University School of Medicine Hangzhou City China; ^4^ Department of Radiotherapy The First Affiliated Hospital Zhejiang University School of Medicine Hangzhou City China

**Keywords:** Glut‐1, HIF‐1α, hypoxic radioresistance, laryngeal carcinoma, PI3K/Akt/mTOR pathway

## Abstract

Hypoxic resistance is the main obstacle to radiotherapy for laryngeal carcinoma. Our previous study indicated that hypoxia‐inducible factor 1α (HIF‐1α) and glucose transporter 1 (Glut‐1) double knockout reduced tumour biological behaviour in laryngeal carcinoma cells. However, their radioresistance mechanism remains unclear. In this study, cell viability was determined by CCK8 assay. Glucose uptake capability was evaluated by measurement of ^18^F‐fluorodeoxyglucose radioactivity. A tumour xenograft model was established by subcutaneous injection of Tu212 cells. Tumour histopathology was determined by haematoxylin and eosin staining, immunohistochemical staining, and TUNEL assays. Signalling transduction was evaluated by Western blotting. We found that hypoxia induced radioresistance in Tu212 cells accompanied by increased glucose uptake capability and activation of the PI3K/Akt/mTOR pathway. Inhibition of PI3K/Akt/mTOR activity abolished hypoxia‐induced radioresistance and glucose absorption. Mechanistic analysis revealed that hypoxia promoted higher expressions of HIF‐1α and Glut‐1. Moreover, the PI3K/Akt/mTOR pathway was a positive mediator of HIF‐1α and/or Glut‐1 in the presence of irradiation. HIF‐1α and/or Glut‐1 knockout significantly reduced cell viability, glucose uptake and PI3K/Akt/mTOR activity, all of which were induced by hypoxia in the presence of irradiation. *In vivo* analysis showed that knockout of HIF‐1α and/or Glut‐1 also inhibited tumour growth by promoting cell apoptosis, more robustly compared with the PI3K inhibitor wortmannin, particularly in tumours with knockout of both HIF‐1α and Glut‐1. HIF‐1α and/or Glut‐1 knockout also abrogated PI3K/Akt/mTOR signalling transduction in tumour tissues, in a manner similar to wortmannin. HIF‐1α and/or Glut‐1 knockout facilitated radiosensitivity in laryngeal carcinoma Tu212 cells by regulation of the PI3K/Akt/mTOR pathway.

## 1 INTRODUCTION

Despite advances in therapy, patients with locally advanced laryngeal carcinoma often experience locoregional, distant recurrence and poor survival.^1^ Radiochemotherapy is important for treatment of these patients.^2^ However, some patients with advanced laryngeal carcinoma may be resistant to radiotherapy, thus causing therapeutic failure.^3^ There remains controversy regarding the best approach for effectively increasing radiosensitivity in laryngeal carcinoma. However, the mechanism of radioresistance in laryngeal carcinoma remains unclear.

A hypoxic microenvironment contributes to radioresistance in laryngeal carcinoma.^4^ HIF‐1α is a vital hypoxic marker and a key transcription factor that mediates cellular adaptation to a hypoxic microenvironment.^5^ Under hypoxia, overexpression of HIF‐1α may increase the invasiveness of cancer cells,^6^ promote distant metastasis^7^, ^8^ and facilitate radioresistance.^9^, ^10^, ^11^, ^12^, ^13^ In previous studies, we found that HIF‐1α overexpression was associated with lymph node metastasis, T stage and poor outcome in patients with laryngeal carcinoma.^14^ Furthermore, HIF‐1α overexpression in laryngeal carcinoma caused radioresistance in AMC‐HN3 cells exposed to hypoxia.^15^ Thus, HIF‐1α regulation may mediate reversal of the effects of hypoxia and provide a novel therapeutic strategy to enhance radiosensitivity in laryngeal carcinoma.

Hypoxia can switch from oxidative phosphorylation to glycolysis.^16^ Under hypoxia, HIF‐1α may enhance expression of angiogenesis‐related genes (e.g. vascular endothelial growth factor) and glycolytic factors (e.g. glucose transporter (Glut)).^17^ Concomitant elevated expression of Glut‐1 leads to enhanced glucose absorption and upregulation of glycolysis.^18^ Under anoxic conditions, overexpression of Glut‐1 enables cancer cells to meet their energy needs via the Warburg effect.^19^ Our previous studies demonstrated that high expression of Glut‐1 is associated with radioresistance in laryngeal carcinoma.^20^, ^21^, ^22^ Additionally, we revealed a significant correlation between HIF‐1α and Glut‐1 in patients with laryngeal carcinoma.^10^, ^14^ Therefore, we presume that HIF‐1α and Glut‐1 act synergistically to induce radioresistance in laryngeal carcinoma. Some studies have shown greater improvement in radiosensitivity by inhibiting multiple genes or targets, compared with knockout of a single gene. With the development of the clustered regularly interspaced short palindromic repeat (CRISPR)/CRISPR‐associated protein 9 (CAS9) genome editing system, two or more genes can be permanently modified and knocked out simultaneously, thus yielding stable inherited mutant cell lines or experimental animals. In a previous study, we confirmed that CRISPR/CAS9‐mediated Glut‐1 and HIF‐1α double knockout robustly reduced the proliferation, migration and invasion of laryngeal carcinoma HEp‐2 cells, possibly by modulating the PI3K/Akt signalling pathway.^23^ However, it remains unclear whether double knockout of Glut‐1 and HIF‐1α using the CRISPR/CAS9 technique affects radiosensitivity in laryngeal carcinoma.

In the present study, we investigated the effects of radiation combined with an agonist or antagonist of the PI3K/Akt pathway, on the expression levels of Glut‐1 and HIF‐1α in laryngeal carcinoma Tu212 cells exposed to normoxic or anoxic conditions. We also investigated whether double knockout of Glut‐1 and HIF‐1α in Tu212 cells contributed to radiosensitivity in those cells and explored the underlying mechanism.

## 2 MATERIALS AND METHODS

This study was approved by the Institutional Review Board of The First Affiliated Hospital, College of Medicine, Zhejiang University, China (No.2020–1550). All authors had access to the study data and reviewed and approved the final manuscript.

### 2.1 Cell culture and treatment

Tu212 cells were purchased from the Chinese Academy of Sciences. Cells were cultured in RPMI‐1640 (Gibco‐BRL) supplemented with 10% heat‐inactivated foetal bovine serum (Hyclone), 2 mM L‐glutamate, 100 U/ml penicillin and 100 g/ml streptomycin at 37°C in a 5% CO_2_ atmosphere. Tu686 cells were purchased from the Chinese Academy of Sciences. Cells were cultured in RPMI‐1640 (Gibco‐BRL) supplemented with 10% heat‐inactivated foetal bovine serum (Hyclone), 100 U/ml penicillin and 100 g/ml streptomycin at 37°C in a 5% CO_2_ atmosphere. The cells were pre‐treated with or without an antagonist (1 mM wortmannin, sc‐3505, Santa Cruz Biotechnology Inc.) or agonist (100 ng/ml insulin‐like growth factor (IGF)‐1, ab270062, Abcam) of PI3K for 24 h, followed by treatment with 12 Gy X‐ray irradiation for an additional 3 h. All cells were then incubated in growth factor‐deprived medium under hypoxic conditions (1% O_2_ by N_2_ displacement, 5% CO_2_) or normoxic conditions (20% O_2_, 5% CO_2_) for an additional 48 h. Single‐energy X‐ray irradiation of 6 MV with a dose rate of approximately 500 MU/min was administered using a linear accelerator (Clinac 23EX, Varian Medical Systems). The plate distance was 100 cm, and the radiation field was 35 × 35 cm. After irradiation, cells were harvested for subsequent experiments. All assays were performed in triplicate.

### 2.2 Double knockout cell model

Single guide RNAs (sgRNAs) targeting HIF‐1α (GAACTCACATTATGTGGAAGTGG and GTTGATAAGGCCTCTGTGATGAGG) and Glut‐1 (GGATGCTCTCCCCATAGCGGTGG and GCCACCACGCTCACCACGCTCTGG) were designed based on the online tool http://crispr.mit.edu/ (CRISPR Design of Massachusetts Institute of Technology) and http://www.e‐crisp.org/E‐CRISP/index.html (E‐Crisp German Cancer Research Center). The sgRNAs were then inserted into the pUC57‐T7‐gRNA plasmid using the BasI restriction enzyme and DNA ligase. Following sequence verification, recombinant plasmids encoding sgRNAs against HIF‐1α and Glut‐1 were transfected (separately and in combination) into Tu212 cells using Lipofectamine 2000 (Invitrogen). After 24 h, the transfected cells were digested, harvested and resuspended at a concentration of 10 cells/ml. A 100‐μl aliquot of the cell suspension was seeded into a 96‐well plate at a density of 1 cell/well, and positive cells were screened. The single cells were then digested in the original well or transferred to a larger culture plate (96‐well plate →24‐well plate →6‐well plate →6‐cm culture dish →10‐cm culture dish), where they formed a sphere. Knockout efficiency was verified in a previous study by our group.^23^ As described above, Tu212 cells transfected with Glut‐1 and HIF‐1α sgRNAs, or both, were treated with 12 Gy X‐ray irradiation for 3 h and then incubated for 48 h under hypoxic or normoxic conditions. All cells were used for subsequent assays.

### 2.3 Glucose uptake *in vitro*

Normal cells or Glut‐1 and/or HIF‐1α knockout cells were incubated under hypoxic or normoxic conditions. After treatment with or without 12 Gy X‐ray irradiation, cells were incubated with medium containing ^18^F‐fluorodeoxyglucose (FDG) for an additional 3 h. They were then digested and collected. The intracellular (C_in_) and extracellular (C_out_) radioactivity levels were determined using a gamma radioimmunoassay counter (WIZARD2, PerkinElmer). The ratio of C_in_ to C_out_ was used as a measure of glucose uptake by tumour cells.

### 2.4 CCK8 assay

Briefly, 1*10^4^ normal cells or Glut‐1 and/or HIF‐1α knockout cells were seeded into each well of 96‐well plate. Cells were treated with 12 Gy for 3 h, followed by incubation for an additional 48 h in a 96‐well plate. Subsequently, all cell groups were incubated with 20 µl cell counting solution (CCK8) for 1 h in darkness. Absorption at 450 nm was then measured using the Spectra Plus microplate reader (Molecular Devices).

### 2.5 EDU staining detection

After the completion of EdU labelled cells (EdU staining kit, Biyuntian, C0078s), the culture medium was removed, and the cells were added with 1m l4% paraformaldehyde. The cells were fixed at room temperature for 15 min. The fixation solution was removed, and each well was washed 3 times with 1ml washing solution. Wash solution was removed, and each well was incubated with 1ml PBS containing 0.3% TritonX‐100 at room temperature for 15 min. 10 ml Click reaction solution was prepared. Each well was added with 0.5 ml of Click reaction at room temperature for 30 min. Click reaction solution was removed, and each well was washed for 3 times. Each well was added with 1ml 1X Hoechst 33342 (Biyuntian, C1025) solution in the dark for 10 min. 1X Hoechst 33342 solution was suck up. Then, the slides were washed twice with PBS and coverslipped with anti‐fluorescence quenching agent. The images were observed and collected under a fluorescence microscope.

### 2.6 Western blotting

Total proteins were extracted from cells and tumour tissues by radioimmunoprecipitation assay lysis buffer. Subsequently, equal amounts of protein (30 μg) were separated by sodium dodecyl sulphate–polyacrylamide gel electrophoresis. After the proteins had been transferred to polyvinylidene difluoride membranes (cat. no. IPVH00010; Millipore), the membranes were blocked with 5% skim milk diluted in Tris‐buffered saline with Tween 20. Membranes were then incubated with primary antibodies against caspase3(1:1000;cat. no. ab32351; Abcam), Glut‐1 (1:200; cat. no. ab40084; Abcam), PI3K (1:1000; cat. no. 4292, Cell Signaling Technology), phosphorylated (p)‐PI3K (1:800; cat. no. 4228, Cell Signaling Technology), p‐Akt (1:1000; cat. no. 4060, Cell Signaling Technology), Akt (1:2000; cat. no. 9272, Cell Signaling Technology), HIF‐1α (1:800; cat. no. Ab1, Abcam), mTOR (1:1000; cat. no. Ab134903, Abcam) and p‐mTOR (1:1000; cat. no. ab109268, Abcam) at 4°C overnight. A primary antibody against GAPDH (1:4000; cat. no. AP0063; Bioworld) served as the internal control. The membranes were followed by incubation with secondary antibodies for 1 h at room temperature. Membranes were stained using an ECL chemiluminescence assay kit (Beyotime Institute of Biotechnology) and analysed semi‐quantitatively using the ChemiDoc XRS+System (Bio‐Rad).

### 2.7 Xenograft model

Six‐ to eight‐week‐old male athymic BALB/c nude mice were purchased from Beijing Vital River Laboratory Animal Technology Co. Ltd. and housed in a specific pathogen‐free room under controlled temperature (20 ± 2°C) and humidity. All animal studies were approved by the Animal Welfare Committee of our research organization (approval number: 2020–1550). After mice had been fed ad libitum for 1 week, approximately 0.2 ml (2 × 10^7^/ml) Tu212 cells were inoculated subcutaneously into the right flank of each mouse. The animal groups were as follows: Control (normal Tu212), 12 Gy (Tu212 + 12 Gy), 12 Gy +wortmannin (Tu212 + 12 Gy +wortmannin), HIF‐1α^−/−^, HIF‐1α^−/−^+12 Gy, Glut‐1^−/−^, Glut‐1^−/−^+12 Gy, HIF‐1α^−/−^ + Glut‐1^−/−^, and HIF‐1α^−/−^ + Glut‐1^−/−^+12 Gy. When the tumour volume reached 100 mm^3^, mice were treated with 12 Gy X‐ray irradiation at 5‐day intervals. X‐ray radiation was performed in the same manner used in the *in vitro* assay. During model establishment, mice received intraperitoneal administration of 1 mg/kg wortmannin, once daily. On Day 24 after the initial injection of cancer cells (Day 17 after X‐ray irradiation), mice were killed by cervical dislocation and imaged using a camera. Tumours in each mouse were then excised, photographed and harvested for subsequent experiments. Tumour volume was calculated according to the following formula: V = ½ × a^2^ × b, where a is the short axis and b the long axis. After weighing, tumour tissues were stored at −80℃ for subsequent experiments.

### 2.8 TUNEL assay

Paraffin‐embedded tumour tissue sections from each group were assessed using the In Situ Cell Death Detection Kit‐POD (Roche). In brief, 5‐μm‐thick tissue sections were dewaxed in xylene and rehydrated in an ethanol gradient. The sections were incubated with 20 μg/ml DNase‐free proteinase K for 15 min at 37°C and washed three times using phosphate‐buffered saline. After blocking using peroxidase for 20 min, the sections were incubated with 50 μl TUNEL reaction mixture for 60 min at 37°C in darkness. Each section was incubated with 50 μl streptavidin–horseradish peroxidase conjugate and stained with 200 μl DAB solution. After mounting, the staining results were observed and photographed by fluorescence microscopy. Ten high‐powered fields were examined for each group. Total cell number were conducted by counting the number of nuclei. The apoptosis cells were conducted by counting the number of green‐labelled nuclei. The proportion of apoptosis cells were analysed by recording the ratio of green‐labelled cell number to total cell number. Double blind was used in the experiment. The analysis was performed by three independent researchers.

### 2.9 Immunohistochemistry

Tumour tissues were collected, fixed in 4% paraformaldehyde for 24 h at 4°C and embedded in paraffin. After 5 µm thick sections had been deparaffinized in xylene and hydrated in gradient ethanol, they were subjected to antigen retrieval using a high‐pressure method. Endogenous peroxidases were blocked by 3% H_2_O_2_ for 10 min at room temperature. Subsequently, sections were incubated with a primary antibody against Ki67 (cat. no. 12202, Cell Signaling Technology) diluted in working buffer (1:50) at 4°C overnight. The next day, all sections were hybridized with a secondary antibody labelled with streptavidin–horseradish peroxidase at 4°C for 50 min Subsequently, the sections were stained with DAB staining buffer, followed by haematoxylin for 25 s. The staining results were visualized by microscopy (Olympus BX41; Olympus Corporation). Positive cells labelled with brown–yellow granules in five random high‐magnification fields of each group were identified and counted by three independent pathologists. After counting the number of total cells (blue‐labelled nuclei), the proportion of Ki67‐positive cells was calculated. Cell number was also monitored by using Image‐J software using the double‐blind method. Means of different tumour tissues were used to generate the histogram.

### 2.10 Haematoxylin and eosin staining

Tumour tissue samples were placed in 4% paraformaldehyde for 48 h at 4°C, dehydrated in gradient ethanol, immersed in xylene and embedded in paraffin. 5 µm thick sections were dewaxed, rehydrated and stained with haematoxylin (Beyotime Institute of Biotechnology) for 5 min and distinguished using ammonium hydroxide. Subsequently, the sections were incubated in eosin solution for 2 min, washed with running water and dewaxed using xylene. Then, sections were mounted with neutral balsam and observed using an optical microscope (Olympus IX71; Olympus Corporation).

### 2.11 Glucose uptake *in vivo*

On Day 24 after the initial injection of cancer cells (Day 17 after X‐ray irradiation), each mouse was injected with 11.1 MBq (300μCi) of ^18^F‐FDG through tail vein. The mice were sacrificed for 1h, and the tumour tissue was stripped to detect its radioactivity by a gamma radioimmunoassay counter (WIZARD2, PerkinElmer). The relative radioactivity of each group was calculated: relative Fold change of control.

### 2.12 Statistical analysis

All independent experiments were performed at least three times. Data are presented as means ± standard error. Data were analysed using IBM SPSS Statistics 25.0 (IBM Corp.) and GraphPad Prism 6.0 (GraphPad Software Inc.) using one‐way analysis of variance. *P* < 0.05 was considered to indicate statistical significance.

## 3 RESULTS

### 3.1 Hypoxia induces radioresistance in Tu212 cells by potentially accelerating glucose uptake

**FIGURE 1:**
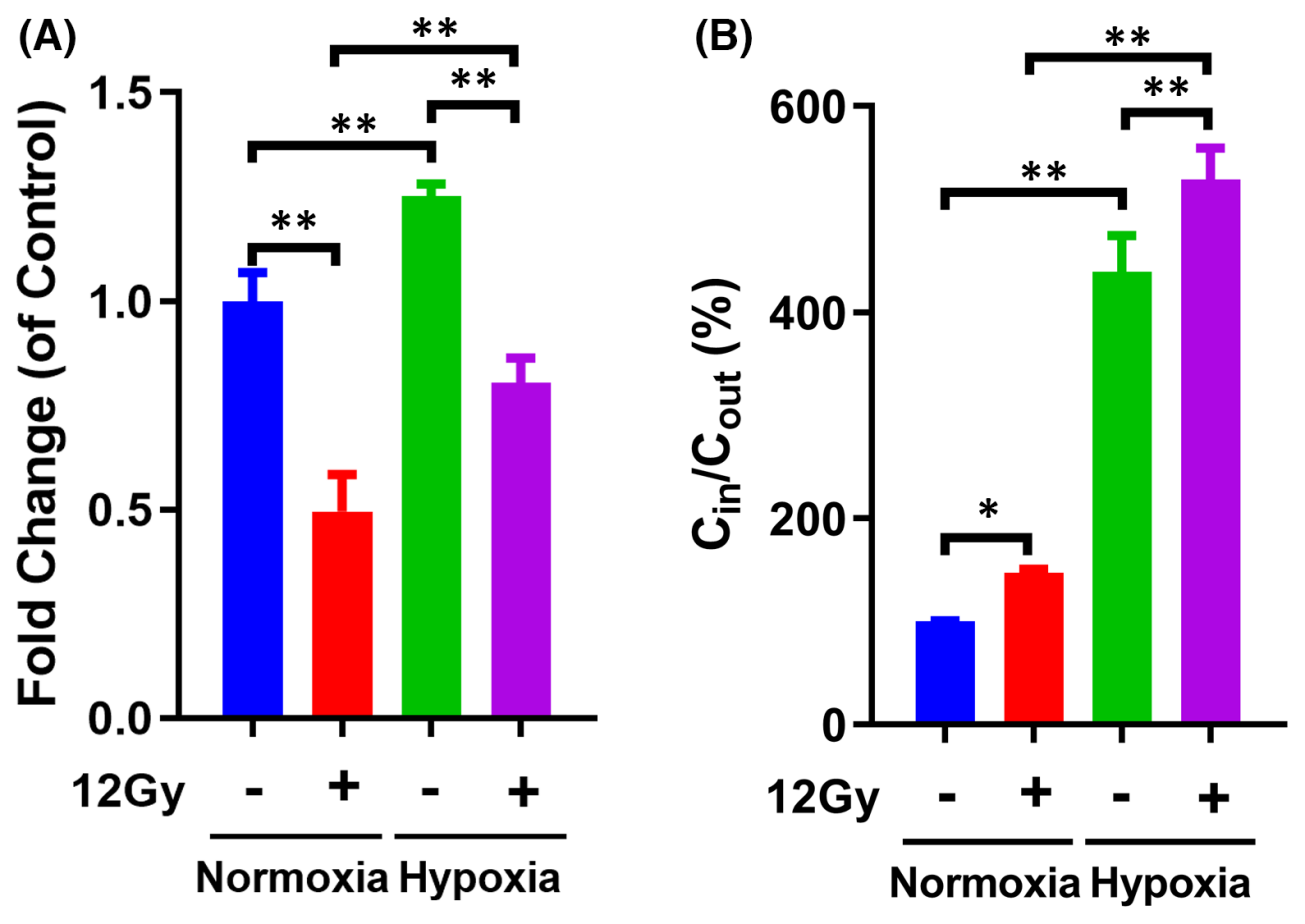
Effects of hypoxia on cell viability and glucose uptake in Tu212 cells exposed to irradiation. A, Tu212 cells were cultured under normoxic and hypoxic conditions, respectively. Cells were then treated with or without 12 Gy X‐ray irradiation for 3 h. Cell viability was determined by CCK8 assay. B, Cells were cultured with medium containing ^18^F‐FDG for an additional 3 h. After irradiation exposure under normoxic or hypoxic conditions, the C_in_ and C_out_ were determined using a gamma radioimmunoassay counter. *: *p* < 0.05; ** and ##: *p* < 0.01

Cancer cells exhibit continuously increasing glucose consumption, even in the presence of oxygen, due to the Warburg effect.^24^ Another cell progression phenomenon, the Pasteur effect, suggests that hypoxia is a leading cause of elevated glucose absorption by tumour cells.^25^, ^26^ Under normoxic conditions, the viability of Tu212 cells decreased significantly, by 50%, after exposure to 12 Gy X‐ray irradiation. Enhanced cell viability was observed in Tu212 cells cultured under hypoxic conditions, compared with those cultured under normoxic conditions. Notably, Tu212 cells cultured under hypoxic conditions exhibited greater cell viability compared with those cultured under normoxic conditions following treatment with 12 Gy X‐ray irradiation (Figure 1A). In addition, the results of EDU showed that radiotherapy could inhibit the laryngeal carcinoma cell proliferation, whereas the cell proliferation was increased under hypoxic conditions. Moreover, the proliferation of Tu212 cells treated with radiotherapy under hypoxic conditions was stronger than that treated with radiotherapy under normoxic conditions (Figure S1A). The results of Western blot demonstrated a consistent trend, that is, radiotherapy could promote the apoptosis‐related protein caspase3 shear in Tu212 cells, and the caspase3 shear ability of Tu212 cells treated with radiotherapy under hypoxia was weaker than that of the radiotherapy group treated with normoxia (Figure S1B). These results suggested that hypoxia could enhance the radioresistance of laryngeal carcinoma cells. To determine whether hypoxia affects radiosensitivity in Tu212 cells by regulation of glucose uptake, a hypoxic radiotracer ^18^F‐FDG was incubated with Tu212 cells, and the C_in_ and C_out_ were measured. The C_in_/C_out_ ratio was regarded as an indicator of glucose uptake by tumour cells. Cells exposed to 12 Gy under normoxic conditions showed moderate elevation of glucose uptake. However, a robust increase in glucose absorption was observed under hypoxic conditions. Additionally, 12 Gy exposure further promoted glucose absorption under hypoxic conditions compared with normoxic conditions (Figure 1B). These results suggest that hypoxia enhanced cell viability and glucose uptake, followed by reduced radiosensitivity, in Tu212 cells. Hypoxia may reduce radiosensitivity in Tu212 cells by affecting glucose uptake and glycolysis.

### 3.2 Hypoxia activates the PI3K/Akt/mTOR pathway

**FIGURE 2:**
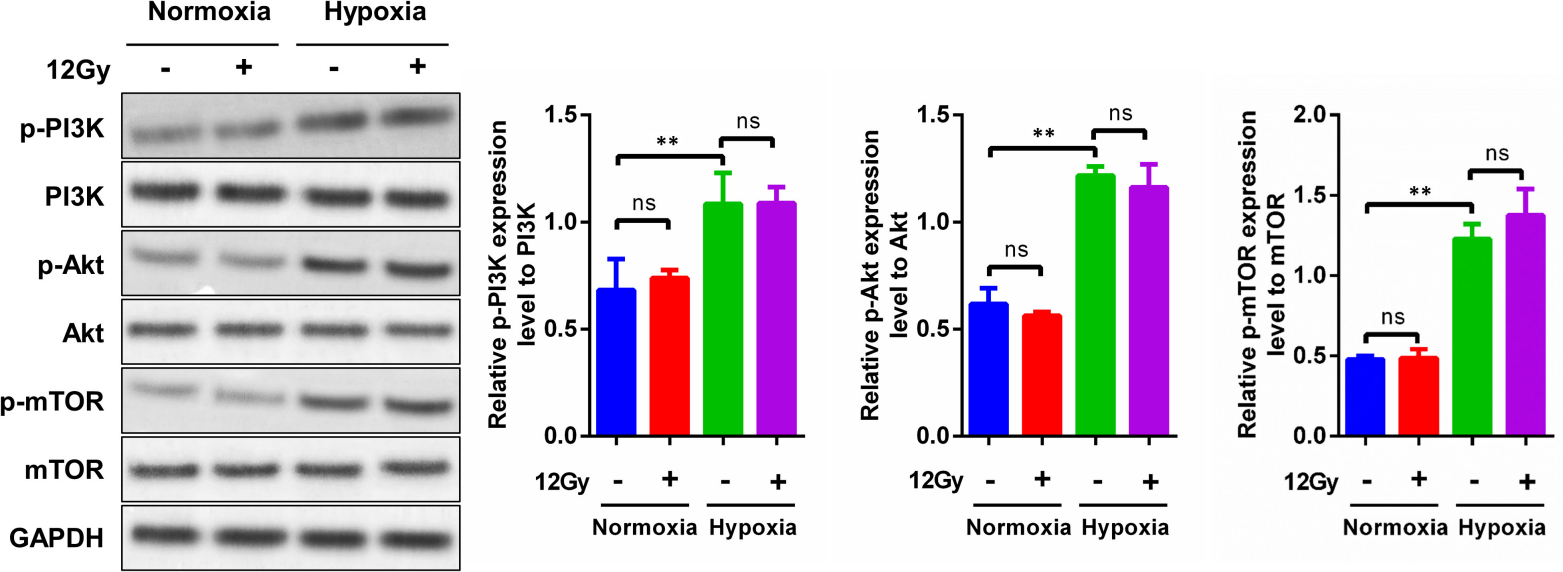
Effects of hypoxia on the PI3K/Akt/mTOR pathway in Tu212 cells. Cells were cultured under normoxic or hypoxic conditions, with or without exposure to 12 Gy X‐ray irradiation. After cell harvest, the protein levels of PI3K, Akt, mTOR, phosphorylated PI3K, phosphorylated Akt and phosphorylated mTOR were determined by Western blotting. GAPDH served as the internal control. Relative quantification of p‐PI3K/PI3K, p‐AKT/AKT and p‐mTOR/mTOR as shown in the right panel. **, *p* < 0.01

Western blotting showed elevated levels of p‐PI3K, p‐Akt and p‐mTOR in Tu212 cells under hypoxic conditions compared with normoxic conditions. These results implied that hypoxia exposure activates the PI3K/Akt/mTOR signalling pathway (Figure 2, third versus first row). However, X‐ray irradiation did not alter the levels of p‐PI3K, p‐Akt or p‐mTOR, regardless of hypoxic or normoxic conditions (Figure 2). These results suggested that hypoxia activated the PI3K/Akt/mTOR pathway, whereas irradiation exposure alone did not.

### 3.3 The PI3K/Akt/mTOR pathway is required for hypoxia‐mediated radioresistance and enhanced expression of Glut‐1 and HIF‐1α

**FIGURE 3:**
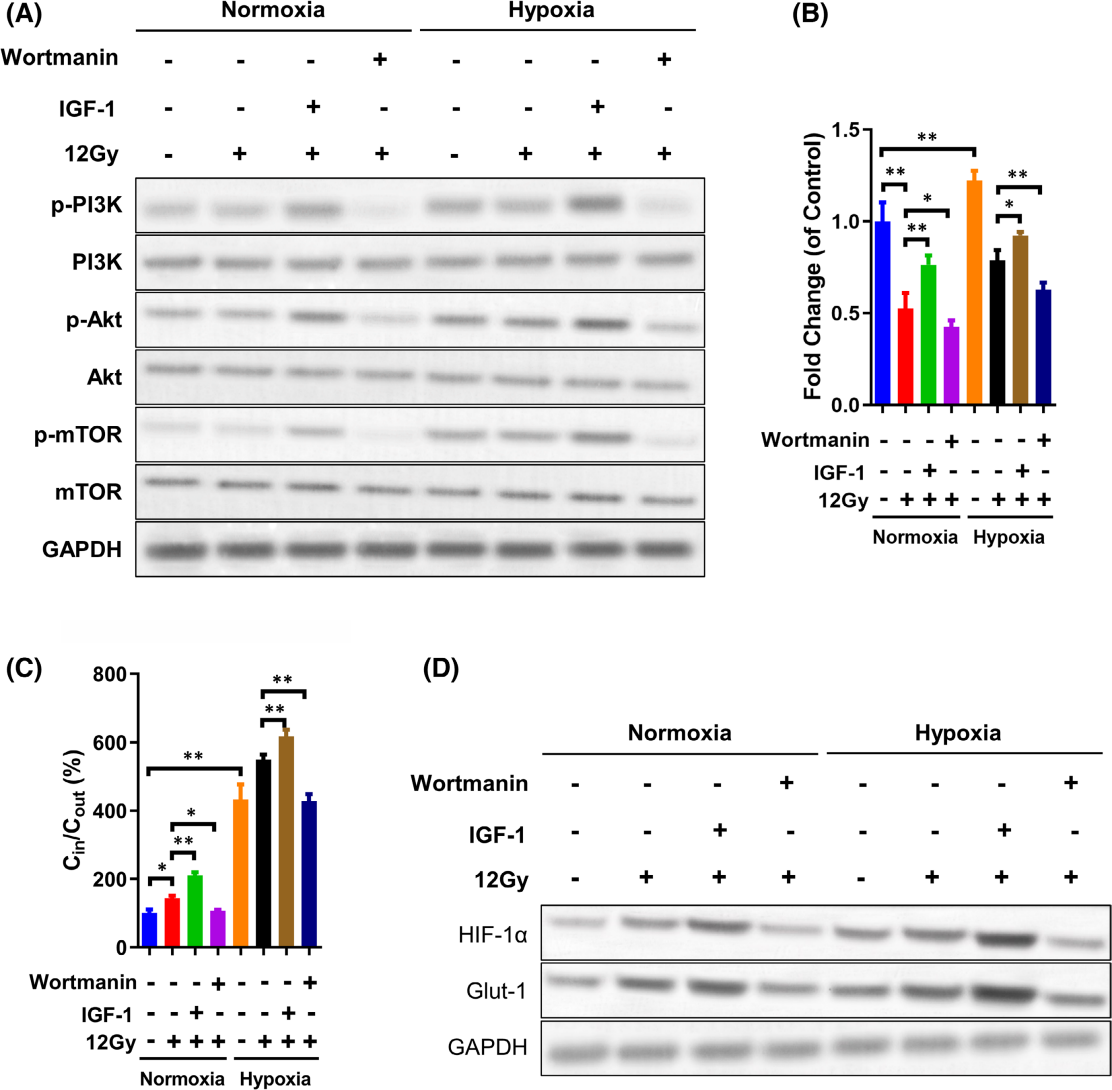
Alterations in Glut‐1 and HIF‐1α protein levels by PI3K inhibition after irradiation exposure under hypoxic conditions in Tu212 cells. A, Cells were cultured under normoxic or hypoxic conditions, with or without exposure to 12 Gy X‐ray irradiation. Protein levels of PI3K, Akt, mTOR, phosphorylated PI3K, phosphorylated Akt and phosphorylated mTOR were determined by Western blotting. B, For cells shown in panel A, viability was measured by CCK8 assays. C, Cells were cultured with medium containing ^18^F‐FDG for an additional 3 h. The C_in_ and C_out_ in each group were measured using a gamma radioimmunoassay counter. D, For cells shown in panel C, the protein levels of Glut‐1 and HIF‐1α were evaluated by Western blotting. Protein quantitative analysis could be found in Figure S2. *, #, and @: *p* < 0.05; **, ##, @@, and &&: *p* < 0.01

We previously demonstrated that the PI3K/Akt pathway contributes to chemo‐radioresistance in laryngeal carcinoma.^20^, ^21^, ^23^, ^27^, ^28^ However, it remains unclear whether the PI3K/Akt pathway facilitates radioresistance in laryngeal carcinoma cells under hypoxic conditions. Thus, wortmannin (an inhibitor of PI3K) and IGF‐1 (an agonist of PI3K) were employed to explore this aspect of radioresistance. As shown in Figure 3A, wortmannin administration significantly diminished, whereas IGF‐1 administration significantly enhanced, PI3K/Akt/mTOR pathway activity in Tu212 cells under normoxic conditions. The levels of p‐PI3K, p‐Akt and p‐mTOR were higher under hypoxic than normoxic conditions. Further treatment with wortmannin reduced PI3K/Akt/mTOR activity, whereas treatment with IGF‐1 enhanced this activity (Figure 3A and Figure S2A). In addition to alteration of the PI3K/Akt/mTOR pathway, IGF‐1 enhanced Tu212 cell viability and proliferation, and inhibit caspase3 shear under hypoxic conditions following 12 Gy X‐ray irradiation, whereas wortmannin aggravated the irradiation‐induced decrease in cell viability and proliferation, and increasing of caspase3 shear under normoxic conditions (Figure 3B and Figure S3). In contrast, the reduced cell viability and proliferation, and increased caspase3 shear induced by 12 Gy X‐ray irradiation were moderately reversed by IGF‐1 and enhanced by wortmannin under hypoxic conditions (Figure 3B and Figure S3). Accordingly, IGF‐1 promoted, whereas wortmannin inhibited, FDG uptake by Tu212 cells after 12 Gy X‐ray irradiation under normoxic or hypoxic conditions (Figure 3C). Moreover, the C_in_/C_out_ ratio was higher under hypoxic or normoxic conditions after IGF‐1 treatment but lower after wortmannin treatment, suggesting that glucose uptake was regulated by the PI3K/Akt/mTOR signalling pathway (Figure 3C).

In the present study, we investigated the expression levels of Glut‐1 and HIF‐1α in X‐ray‐irradiated laryngeal carcinoma under hypoxic conditions. The protein levels of Glut‐1 and HIF‐1α in laryngeal carcinoma Tu212 cells were significantly elevated under hypoxic conditions compared with normoxic conditions. Additionally, 12 Gy X‐ray irradiation enhanced the protein levels of Glut‐1 and HIF‐1α and amplified the hypoxia‐induced increases in the levels of both proteins (Figure 3D and Figure S2B). PI3K activation was elevated by IGF‐1 but inactivated by wortmannin. These effects altered the levels of Glut‐1 and HIF‐1α in the presence of 12 Gy X‐ray irradiation, regardless of exposure to normoxic or hypoxic conditions (Figure 3D and Figure S2B). These data suggested that hypoxia promoted Glut‐1 and HIF‐1α expression, presumably via the PI3K/Akt/mTOR pathway, followed by increased glucose uptake and radioresistance in Tu212 cells.

### 3.4 Knockout of Glut‐1 and HIF‐1α increases radiosensitivity in Tu212 cells via the PI3K/Akt/mTOR pathway

**FIGURE 4:**
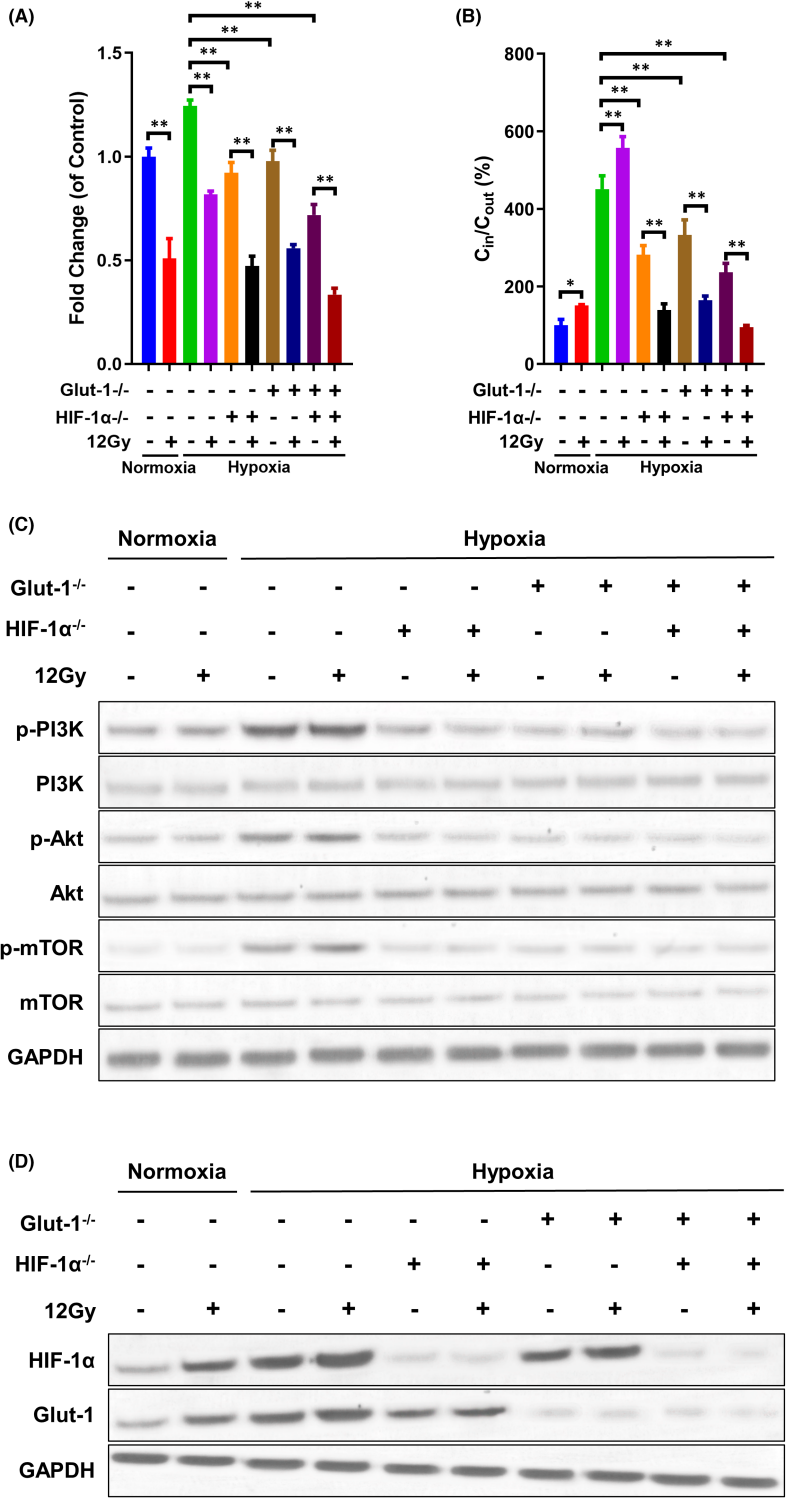
Effects of Glut‐1 and HIF‐1α knockout on radioresistance and the PI3K/Akt/mTOR pathway activity in Tu212 cells. A, Glut‐1 and/or HIF‐1α knockout was performed in Tu212 cells. Subsequently, normal cells were exposed to irradiation under normoxic conditions, while normal cells or Glut‐1 and/or HIF‐1α knockout cells were cultured under hypoxic conditions. Cell viability in each group was measured using CCK8 assays. B, Cells were treated under normoxic or hypoxic conditions, with or without exposure to 12 Gy X‐ray irradiation. The C_in_ and C_out_ in each group were measured using a gamma radioimmunoassay counter. In the above cell groups, protein levels of PI3K, Akt, mTOR, phosphorylated PI3K, phosphorylated Akt, phosphorylated mTOR (C), as well as Glut‐1 and HIF‐1α (D) were determined by Western blotting. *: *p* < 0.05; **: *p* < 0.01

Our previous study demonstrated that Glut‐1 and HIF‐1α double knockout inhibited FDG uptake by HEp‐2 cells, leading to reduced viability.^23^ We further investigated whether knockout of the two genes contributed to radioresistance under hypoxic conditions. Consistent with the previous findings, 12 Gy X‐ray irradiation led to reduced cell viability and proliferation and elevated caspase3 shear and glucose uptake in Tu212 cells under normoxic or hypoxic conditions, although cell viability, proliferation and glucose absorption were greater, cleaved‐caspase3 expression was lower under hypoxic conditions than under normoxic conditions (Figure 4A,B and Figure S4). The hypoxia‐induced increases in cell viability and proliferation, glucose uptake, and decreases the expression of cleaved‐caspase3 expression were reversed by Glut‐1 or HIF‐1α knockout. The lowest cell viability and proliferation, glucose uptake, and the highest contents of cleaved‐caspase3 were observed in Glut‐1 and HIF‐1α double knockout cells without 12 Gy X‐ray irradiation (Figure 4A,B and Figure S4). During irradiation exposure, Glut‐1 or HIF‐1α single or double knockout further reduced hypoxia‐mediated cell proliferation and glucose uptake, near the levels observed under normoxic conditions, with the lowest levels observed in the double knockout cells (Figure 4A,B and Figure S4A). Moreover, the caspase3 shear level of Tu212 cells treated with GLUT‐1 or HIF‐1α single or double Knockout after radiotherapy under hypoxic conditions was significantly higher than that after radiotherapy under the normoxic conditions. The caspase3 shear level of Tu212 cells by double knockout was the highest (Figure S4B). These findings suggest that Glut‐1 or HIF‐1α knockout improved hypoxia‐induced radioresistance. Furthermore, compared with normoxic conditions, activation of the PI3K/Akt/mTOR pathway under hypoxic conditions was abrogated by Glut‐1 or HIF‐1α knockout (Figure 4C and Figure S5A). Notably, 12 Gy X‐ray irradiation did not affect the levels of p‐PI3K, p‐Akt or p‐mTOR in normal cells or Glut‐1/HIF‐1α knockout cells (Figure 4C and Figure S5A). The efficiency of gene knockout was verified by Western blotting (Figure 4D and Figure S5B). Importantly, Glut‐1 and HIF‐1α knockout regulated each other because Glut‐1 (HIF‐1α) knockout reduced the hypoxia‐induced constitutive expression of HIF‐1α (Glut‐1), regardless of X‐ray irradiation (Figure 4D and Figure S5B). The role of Glut‐1 and HIF‐1α knockout on radiotherapy resistance was verified in another laryngeal carcinoma cell line Tu686 (Figure S6). Collectively, the data demonstrated greater radiosensitivity in Glut‐1/HIF‐1α knockout than normal cells under hypoxic conditions, in addition to inactivation of the PI3K/Akt/mTOR pathway.

### 3.5 Knockout of Glut‐1 and HIF‐1α elevates radiosensitivity in Tu212 cells *in vivo*

**FIGURE 5:**
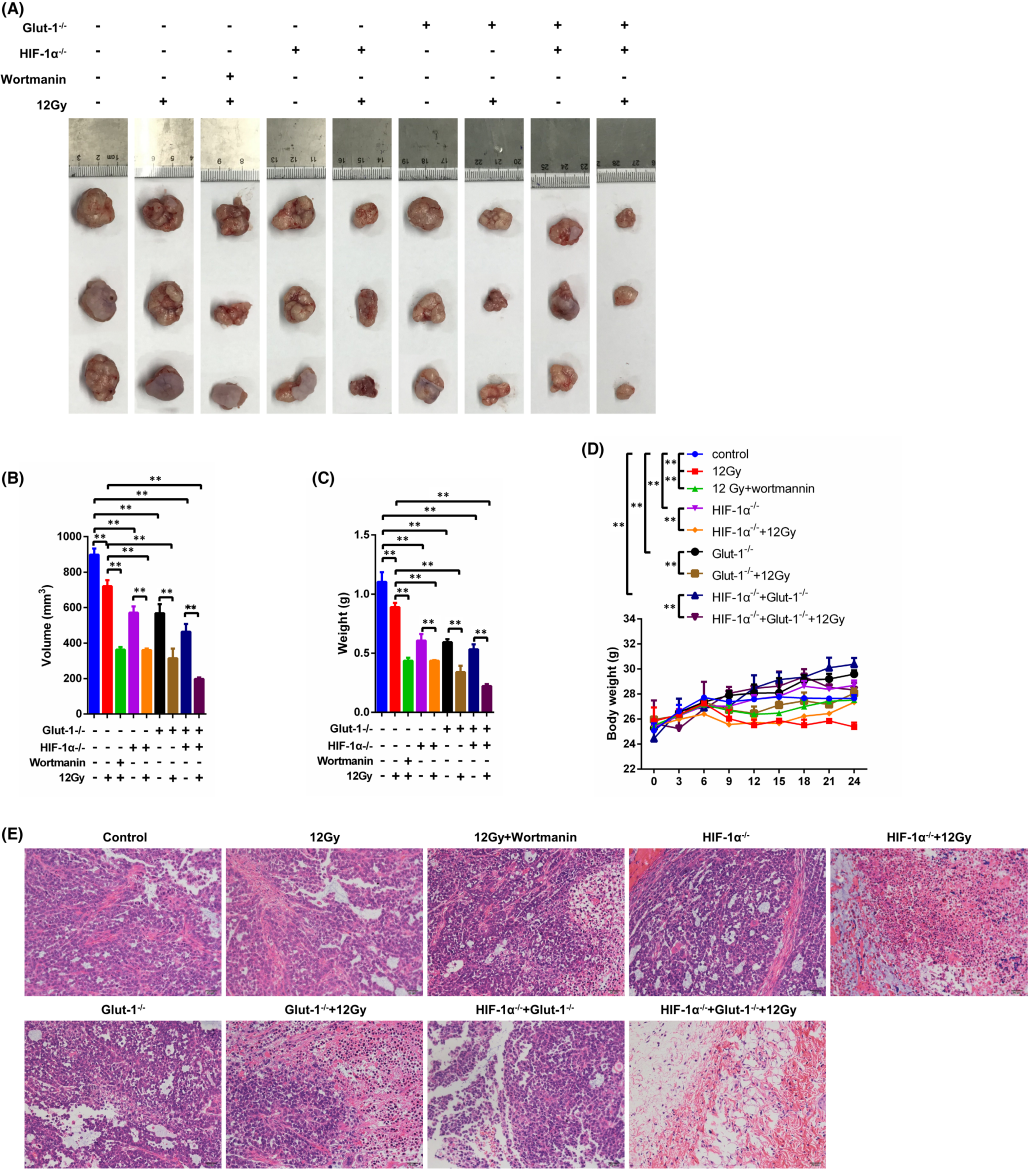
Associations of Glut‐1 and HIF‐1α with radiosensitivity *in vivo*. A, Tu212 cells were injected into the right flank of nude mice. After establishment of tumours, tumour tissues were excised and photographed. B, Tumour volumes were calculated using the following formula: V = 1/2 × a^2^ × b, where a is the short axis and b the long axis. Tumour weight (C) and body weight (D) were analysed in each group. (E) Histopathological analysis of the lesions was performed by haematoxylin and eosin staining. *: *p* < 0.05; **: *p* < 0.01

In the xenograft model, wortmannin administration clearly enhanced irradiation‐induced prevention of tumour growth (Figure 5A), leading to a lower tumour volume and weight (Figure 5B,C). Additionally, knockout of Glut‐1 and/or HIF‐1α limited tumour growth, tumour volume and tumour weight compared with tumours in control mice. These inhibitory effects were slightly greater than those after 12 Gy X‐ray irradiation alone (Figure 5A–C). Moreover, greater radiosensitivity in tumour tissues was observed after knockout of both genes than either gene alone, with tumour growth nearly abolished in double knockout mice following irradiation exposure (Figure 5A–C). With respect to alterations in tumour weight, body weight showed an inverse relationship with tumour weight. Either Glut‐1 or HIF‐1α could elevate body weight, and greater body weight was seen in mice with double knockout of Glut‐1 and HIF‐1α. 12 Gy exposure significantly reduced tumour weight compared to control, Glut‐1/ HIF‐1α knockout group or double knockout group. The lowest body weight was observed in mice with normal tumours following exposure to 12 Gy alone (Figure 5D). HE staining revealed many areas of necrosis in tumours exposed to wortmannin and/or 12 Gy irradiation, as well as in Glut‐1 and/or HIF‐1α knockout tumours. Greater tumour necrosis areas were verified in Glut‐1 and/or HIF‐1α knockout tumours in the presence of irradiation (Figure 5E), indicating that Glut‐1 and/or HIF‐1α knockout improved radiosensitivity in laryngeal carcinoma cells.

### 3.6 Glut‐1 and HIF‐1α knockout promotes irradiation‐induced apoptosis and proliferation inhibition *in vivo*

**FIGURE 6:**
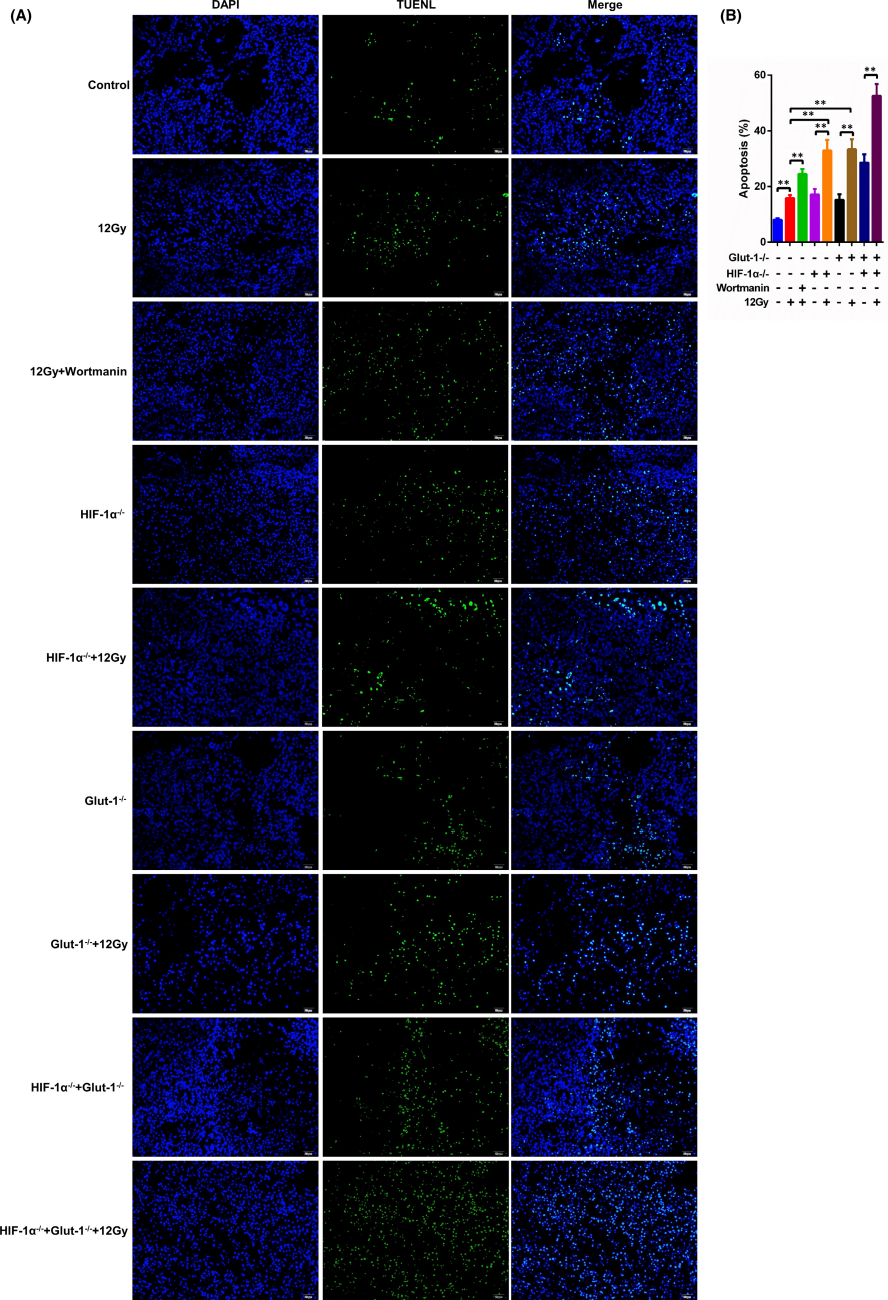
Analysis of apoptosis in tumour tissues. A, Tumour tissues were embedded in paraffin and then stained using TUNEL reaction mixture. Apoptotic cells were observed by fluorescence microscopy. B, The proportion of apoptotic cells was counted. **: *p* < 0.01

We investigated whether the radiosensitivity induced by Glut‐1/HIF‐1α knockout was related to tumour death. Knockout of Glut‐1 or HIF‐1α led to significantly more apoptotic cells in tumour tissues compared with the control, similar to the effects of 12 Gy X‐ray irradiation (Figure 6A,B). PI3K inhibition by wortmannin further increased apoptosis following 12 Gy X‐ray irradiation, similar to the findings in Glut‐1 or HIF‐1α knockout tumours exposed to 12 Gy X‐ray irradiation (Figure 6A,B). As predicted, the highest number of apoptotic cells was observed in the double knockout mice following irradiation exposure (Figure 6A,B). In contrast, few proliferating cells were observed in the presence of 12 Gy X‐ray irradiation or in the absence of Glut‐1 or HIF‐1α (Figure S7A,B). Similar to the effects of wortmannin, Glut‐1 or HIF‐1α knockout enhanced the anti‐proliferative effect of 12 Gy, with the fewest proliferating cells observed in Glut‐1 and HIF‐1α double knockout tumours following irradiation (Figure S7A,B). These results indicated that both inhibition of PI3K and knockout of Glut‐1/HIF‐1α enhanced irradiation‐induced apoptosis and inhibited proliferation.

### 3.7 Glut‐1 and HIF‐1α knockout inhibits the PI3K/Akt/mTOR pathway and glucose uptake *in vivo*

**FIGURE 7:**
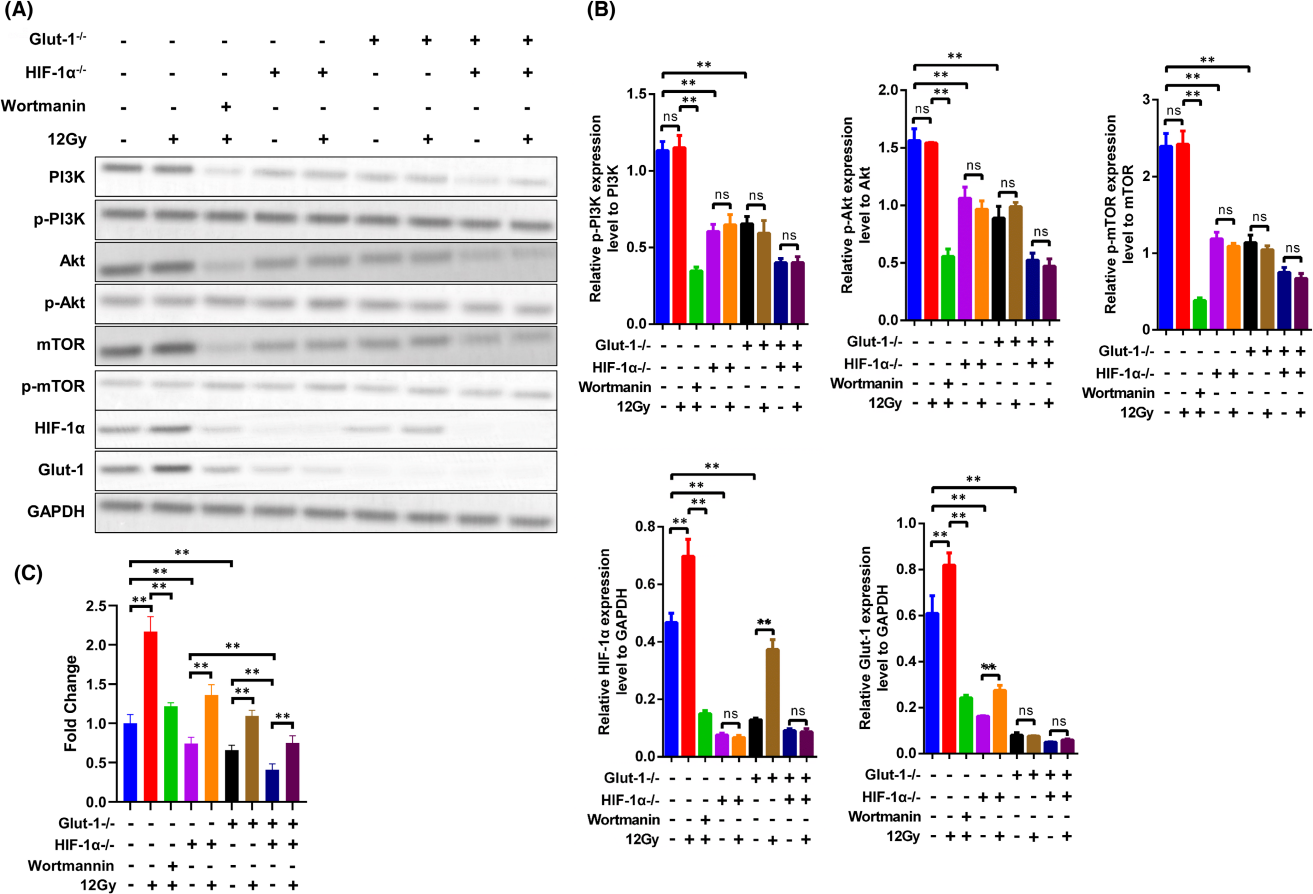
Effects of Glut‐1 and HIF‐1α knockout on PI3K/Akt/mTOR pathway activity and glucose uptake in tumour tissues. Protein levels of PI3K, Akt, mTOR, phosphorylated PI3K, phosphorylated Akt, phosphorylated mTOR, Glut‐1 and HIF‐1α were determined by Western blotting (A). Relative quantification of p‐PI3K/PI3K, p‐AKT/AKT, p‐mTOR/mTOR, HIF‐1α/GAPDH and Glut‐1/GAPDH as shown in the right panel (B). The results of ^18^F‐FDG radioactive of tumour tissues of xenograft(C) **, *p* < 0.01

As mentioned previously, knockout of Glut‐1 and HIF‐1α led to radiosensitivity in Tu212 cells, presumably via the PI3K/Akt/mTOR pathway. Here, 12 Gy X‐ray irradiation did not affect the activities of p‐PI3K, p‐Akt or p‐mTOR, regardless of Glut‐1 and/or HIF‐1α knockout, although it increased Glut‐1 and HIF‐1α protein levels. In addition, wortmannin inhibited the protein levels of Glut‐1 and HIF‐1α. Knockout of Glut‐1 and HIF‐1α further reduced activation of the PI3K/Akt/mTOR pathway (Figure 7A,B). These data were consistent with the *in vitro* results, suggesting positive feedback regulation between the PI3K/Akt/mTOR pathway and Glut‐1/HIF‐1α expression. Therefore, in tumour tissues under hypoxic conditions, activation of the PI3K/Akt/mTOR pathway promoted the expression of Glut‐1 and HIF‐1α. The results of ^18^F‐FDG radioactive of tumour tissues of xenograft that radiotherapy could increase glucose uptake in tumour tissues of xenograft, whereas wortmannin could inhibit glucose uptake in tumour tissues of xenograft (Figure 7C). Knockout of Glut‐1 and HIF‐1α could inhibit glucose uptake in tumour tissues of xenograft, and block the enhanced glucose uptake ability of tumour tissue induced by radiotherapy (Figure 7C). Double knockout treatment had the strongest inhibition effect on the glucose uptake (Figure 7C). The increased expression of Glut‐1 and HIF‐1α activated the PI3K/Akt/mTOR pathway and glucose uptake *in vivo*, thereby leading to radioresistance.

## 4 DISCUSSION

Radiotherapy is an important treatment option for laryngeal carcinoma.^29^ However, the development of radioresistance leads to persistent treatment failure. Hypoxia is an important factor mediating tumour progression and therapeutic resistance, partly via the expression of HIF‐1α.^30^ Hypoxia induces the stabilization of HIF‐1α, which is associated with radiotherapy resistance in head and neck carcinoma.^31^ Our previous research indicated a significant association between Glut‐1 and radioresistance in laryngeal carcinoma.^20^, ^21^, ^22^, ^32^, ^33^ Furthermore, HIF‐1α and Glut‐1 double knockout using the CRISPR/CAS9 system showed synergistic regulation of biological behaviour in laryngeal carcinoma cells.^23^ Glut‐1 antisense oligodeoxynucleotides facilitated the response to radiotherapy in laryngeal carcinoma tissue via autophagy regulation.^32^ Herein, protein levels of HIF‐1α and Glut‐1 were enhanced after 12 Gy X‐ray irradiation, further confirming the associations of HIF‐1α and Glut‐1 with radiotherapy. In the present study, we verified that HIF‐1α and/or Glut‐1 knockout reversed the reduced cell viability induced by irradiation, leading to increased radiosensitivity of Tu212 cells. Thus, Glut‐1 and HIF‐1α knockout contributed to radiosensitivity in laryngeal carcinoma cells. In addition, double knockout of Glut‐1 and HIF‐1α led to greater radiosensitivity in laryngeal carcinoma cells under hypoxic conditions, compared with single gene knockouts. Double knockout of Glut‐1 and HIF‐1α may be a novel strategy for enhancing radiosensitivity in laryngeal carcinoma.

Cancer cells located in hypoxic microenvironments trigger glycolysis in response to the reduced oxygen supply.^34^ Glycolysis elevates the lactate level, leading to a poor clinical outcome in patients with head and neck cancer treated with radiotherapy.^35^ ^18^F‐FDG (glycolysis) uptake is correlated with endogenous markers of hypoxia, glycolysis, and cell proliferation, as ^18^F‐FDG is a good indicator of rapid cell proliferation.^36^ In this study, hypoxia induced radioresistance in laryngeal carcinoma cells, along with enhanced ^18^F‐FDG uptake and an increased C_in_/C_out_ ratio. Thus, following exposure to 12 Gy X‐ray irradiation in Tu212 cells, high cell viability under hypoxic conditions may be more closely related to ^18^F‐FDG uptake and a high rate of glycolysis, indicative of radioresistance. Wogonin can abolish hypoxia resistance in colon cancer cells by reducing the rate of glycolysis and inhibiting PI3K/Akt pathway activity.^37^ Activation of PI3K/Akt reduces glycogen synthesis, resulting in enhanced glycolysis.^38^ Our previous research indicated that a HIF‐1α and Glut‐1 double knockout approach reduced cell proliferation, migration and invasion in a PI3K/Akt/mTOR signalling‐dependent manner.^23^ In this investigation, hypoxia induced activation of the PI3K/Akt/mTOR pathway; inhibition of this pathway abrogated hypoxia‐induced glycolysis and radioresistance in Tu212 cells, whereas hyperactivation enhanced these processes. Insulin‐like growth factor (IGF‐1) has the similar functions with insulin analogue to activate the PI3K‐Akt signalling pathway.^39^ In this study, IGF‐1 treatment activated PI3K‐Akt‐mTOR signalling pathway, enlarged the resistance to radiotherapy, and elevated glucose uptake and the expression of HIF‐1α and Glut‐1 in laryngeal cancer cells. Since IGF‐1 might exert regulatory role on other signalling pathway,^40^ the opposite effects on PI3K‐Akt pathway between wortmannin and IGF‐1 were observed in TU212 and TU686 cells. The data indirectly demonstrated the activation of PI3K‐Akt in IGF‐1‐challenged laryngeal cancer cells, ruling out the off‐target effect. Collectively, our findings indicate that the PI3K/Akt/mTOR pathway is required for the progression of radioresistance in laryngeal carcinoma Tu212 cells undergoing glycolysis due to hypoxia.

When tumour cells adapt to hypoxic stress, cellular pathways controlling glucose uptake are activated by HIF‐1α to facilitate cell proliferation and progression.^7^, ^41^ The presence of hypoxia has been established as an important factor (along with HIF‐1α and Glut‐1 activities) affecting tumour radioresistance.^42^ HIF‐1α and Glut‐1 are two important hypoxic markers associated with radioresistance in laryngeal carcinoma. Our previous research showed a robust association between Glut‐1 and HIF‐1α in laryngeal carcinoma.^14^ Hypoxia‐induced increases in HIF‐1α and Glut‐1 levels were reversed by pineapple pulp treatment.^43^ In the present study, we found that double knockout of HIF‐1α and Glut‐1 led to the strongest anti‐proliferative and pro‐apoptotic effects on Tu212 cells, both *in vitro* and *in vivo*. Additionally, we found a decreased Glut‐1 protein level after knockout of HIF‐1α; the inverse relationship was also present. Potentially, Glut‐1‐mediated glucose uptake involves hypoxia‐induced HIF‐1α expression. The reduction in HIF‐1α induced by Glut‐1 knockout was also found in tumour tissues, and the detailed mechanism requires further investigation.

A previous *in vivo* study showed that apigenin‐mediated reduction of Glut‐1 and inactivation of the PI3K/Akt pathway contributed to radiosensitivity in laryngeal carcinoma.^21^ In contrast, the present study demonstrated a positive feedback loop between the PI3K/Akt/mTOR pathway and expression of HIF‐1α and Glut‐1 after exposure to irradiation and hypoxia. Moreover, HIF‐1α and Glut‐1 protein levels were positively regulated by activation of the PI3K/Akt/mTOR pathway. These results confirmed that crosstalk between HIF‐1α/Glut‐1 and the PI3K/Akt/mTOR signalling pathway facilitated cell viability and glucose uptake during radiotherapy under hypoxic conditions. Notably, the response to radiotherapy was more powerful in HIF‐1α and/or Glut‐1 knockout tumours than in tumours treated with wortmannin only, particularly in tumours with double knockout. A possible explanation is that knockout of HIF‐1α or Glut‐1 directly promotes radiosensitivity while inhibiting activation of the PI3K/Akt/mTOR pathway. These changes led to improved radiotherapeutic effects on laryngeal tumours. These findings were consistent with the results in hypoxic tumour cells *in vitro*. Actually, the expression of HIF‐1α/Glut‐1 and activation of PI3K/Akt/mTOR are contacted, influenced and cause‐and‐effect each other. However, we could distinguish which one activate the other one and which one is another one's upstream factor. Maybe, there is a kind of like X‐factor controlling the both HIF‐1a/Glut‐1 and the PI3K/Akt/mTOR pathway, which might be verified by like single cell sequencing or proteomics in further study.

In conclusion, the findings in this study suggest that both the PI3K/Akt/mTOR signalling pathway and HIF‐1α/Glut‐1 are activated in hypoxic tumour cells, thereby facilitating glucose uptake radioresistance in laryngeal carcinoma cells. HIF‐1α and/or Glut‐1 could be promising candidates for improving radiosensitivity in laryngeal carcinoma.

## CONFLICT OF INTEREST

The authors declare no conflict of interest.

## AUTHOR CONTRIBUTIONS

**Yang‐Yang Bao:** Conceptualization (equal); Writing – original draft (equal). **Jiang‐Tao Zhong:** Data curation (equal); Methodology (equal). **Li‐Fang Shen:** Conceptualization (equal); Methodology (equal). **Li‐Bo Dai:** Resources (equal); Software (equal). **Shui‐Hong Zhou:** Conceptualization (equal); Funding acquisition (lead); Investigation (lead); Methodology (lead); Writing – review & editing (lead). **Jun Fan:** Conceptualization (equal); Data curation (equal); Investigation (equal); Methodology (equal); Validation (equal). **Hong‐Tian Yao:** Investigation (equal); Methodology (equal). **Zhong‐Jie Lu:** Methodology (equal); Resources (equal); Software (equal).

## Supporting information

Fig S1

Fig S2

Fig S3

Fig S4

Fig S5

Fig S6

Fig S7

## Data Availability

The data used to support the findings of this study are available from the corresponding author upon request.

## References

[1] Anschuetz L , Shelan M , Dematté M , Schubert AD , Giger R , Elicin O . Long‐term functional outcome after laryngeal cancer treatment. Radiat Oncol. 2019;14:101. 31186027 10.1186/s13014-019-1299-8 PMC6558792

[2] Iizuka Y , Yoshimura M , Inokuchi H , et al. Recurrence patterns after postoperative radiotherapy for squamous cell carcinoma of the pharynx and larynx. Acta Oto‐Laryngol. 2015;135:96‐102. 10.3109/00016489.2014.949848 25351439

[3] Qureishi A , Rieunier G , Shah KA , et al. Radioresistant laryngeal cancers upregulate type 1 IGF receptor and exhibit increased cellular dependence on IGF and EGF signalling. Clin Otolaryngol. 2019;44:1026‐1036. 31536667 10.1111/coa.13434

[4] Vaupel P , Mayer A . Hypoxia in cancer: significance and impact on clinical outcome. Cancer Metast Rev. 2007;26:225‐239. 10.1007/s10555-007-9055-1 17440684

[5] Palazon A , Goldrath AW , Nizet V , Johnson RS . HIF Transcription Factors, Inflammation, and Immunity. Immunity. 2014;41:518‐528. 25367569 10.1016/j.immuni.2014.09.008 PMC4346319

[6] Wu FX , Gao HY , Liu KG , et al. The IncRNA ZEB2‐AS1 is upregulated in gastric cancer and affects cell proliferation and invasion via miR‐143‐5p/HIF‐1 alpha axis. Oncotargets Ther. 2019;12:657‐667. 10.2147/OTT.S175521 PMC6343511 30705594

[7] Rankin EB , Giaccia AJ . Hypoxic control of metastasis. Science. 2016;352:175‐180. 27124451 10.1126/science.aaf4405 PMC4898055

[8] Klaus A , Fathi O , Tatjana TW , Bruno N , Oskar K . Expression of hypoxia‐associated protein HIF‐1 alpha in follicular thyroid cancer is associated with distant metastasis. Pathol Oncol Res. 2018;24:289‐296. 28474313 10.1007/s12253-017-0232-4

[9] Hu Y , E H , Yu X , et al. Correlation of quantitative parameters of magnetic resonance perfusion‐weighted imaging with vascular endothelial growth factor, microvessel density and hypoxia‐inducible factor‐1 alpha in nasopharyngeal carcinoma: evaluation on radiosensitivity study. Clin Otolaryngol. 2018;43:425‐433. 28892580 10.1111/coa.12982

[10] Shen LF , Zhao X , Zhou SH , et al. In vivo evaluation of the effects of simultaneous inhibition of GLUT‐1 and HIF‐1alpha by antisense oligodeoxynucleotides on the radiosensitivity of laryngeal carcinoma using micro 18F‐FDG PET/CT. Oncotarget. 2017;8:34709‐34726. 28410229 10.18632/oncotarget.16671 PMC5471005

[11] Wang D , Qin Q , Jiang QJ , Wang DF . Bortezomib sensitizes esophageal squamous cancer cells to radiotherapy by suppressing the expression of HIF‐1alpha and apoptosis proteins. J Xray Sci Technol. 2016;24:639‐646. 27080362 10.3233/XST-160571

[12] Hennessey D , Martin LM , Atzberger A , Lynch TH , Hollywood D , Marignol L . Exposure to hypoxia following irradiation increases radioresistance in prostate cancer cells. Urol Oncol‐Semin Ori. 2013;31:1106‐1116. 10.1016/j.urolonc.2011.10.008 22130126

[13] Staab A , Fleischer M , Loeffler J , et al. Small interfering RNA targeting HIF‐1 alpha reduces hypoxia‐dependent transcription and radiosensitizes hypoxic HT 1080 human fibrosarcoma cells in vitro. Strahlenther Onkol. 2011;187:252‐259. 21437769 10.1007/s00066-011-2167-0

[14] Wu XH , Chen SP , Mao JY , Ji XX , Yao HT , Zhou SH . Expression and significance of hypoxia‐inducible factor‐1 alpha and glucose transporter‐1 in laryngeal carcinoma. Oncol Lett. 2013;5:261‐266. 23255932 10.3892/ol.2012.941 PMC3525510

[15] Moon SY , Chang HW , Roh JL , et al. Using YC‐1 to overcome the radioresistance of hypoxic cancer cells. Oral Oncol. 2009;45:915‐919. 19457706 10.1016/j.oraloncology.2009.04.005

[16] Sormendi S , Wielockx B . Hypoxia pathway proteins as central mediators of metabolism in the tumor cells and their microenvironment. Front Immunol. 2018;9:40. 29434587 10.3389/fimmu.2018.00040 PMC5796897

[17] Melstrom LG , Salabat MR , Ding XZ , et al. Apigenin down‐regulates the hypoxia response genes: HIF‐1alpha, GLUT‐1, and VEGF in human pancreatic cancer cells. J Surg Res. 2011;167:173‐181. 21227456 10.1016/j.jss.2010.10.041

[18] Chen J , Cui BM , Fan YP , et al. Protein kinase D1 regulates hypoxic metabolism through HIF‐1 and glycolytic enzymes incancer cells. Oncol Rep. 2018;40:1073‐1082. 29901206 10.3892/or.2018.6479

[19] Zhang TB , Zhao Y , Tong ZX , Guan YF . Inhibition of glucose‐transporter 1 (GLUT‐1) expression reversed Warburg effect in gastric cancer cell MKN45. Int J Clin Exp Med. 2015;8:2423‐2428. 25932183 PMC4402830

[20] Luo XM , Xu B , Zhou ML , et al. Co‐inhibition of GLUT‐1 expression and the PI3K/Akt signaling pathway to enhance the radiosensitivity of laryngeal carcinoma xenografts in vivo. PLoS One. 2015;10:e0143306. 26600164 10.1371/journal.pone.0143306 PMC4658010

[21] Bao YY , Zhou SH , Lu ZJ , Fan J , Huang YP . Inhibiting GLUT‐1 expression and PI3K/Akt signaling using apigenin improves the radiosensitivity of laryngeal carcinoma in vivo. Oncol Rep. 2015;34:1805‐1814. 26238658 10.3892/or.2015.4158

[22] Yan SX , Luo XM , Zhou SH , et al. Effect of antisense oligodeoxynucleotides glucose transporter‐1 on enhancement of radiosensitivity of laryngeal carcinoma. Int J Med Sci. 2013;10:1375‐1386. 23983599 10.7150/ijms.6855 PMC3753417

[23] Lu ZJ , Yu Q , Zhou SH , et al. Construction of a GLUT‐1 and HIF‐1 alpha gene knockout cell model in HEp‐2 cells using the CRISPR/Cas9 technique. Cancer Manag Res. 2019;11:2087‐2096. 30881132 10.2147/CMAR.S183859 PMC6413817

[24] Zhang G , Li J , Wang X , et al. The reverse Warburg effect and 18F‐FDG uptake in non‐small cell lung cancer A549 in mice: a pilot study. J Nucl Med. 2015;56:607‐612. 25722447 10.2967/jnumed.114.148254

[25] Busk M , Horsman MR , Jakobsen S , Bussink J , van der Kogel A , Overgaard J . Cellular uptake of PET tracers of glucose metabolism and hypoxia and their linkage. Eur J Nucl Med Mol Imaging. 2008;35:2294‐2303. 18682937 10.1007/s00259-008-0888-9

[26] Busk M , Horsman MR , Kristjansen PE , van der Kogel AJ , Bussink J , Overgaard J . Aerobic glycolysis in cancers: implications for the usability of oxygen‐responsive genes and fluorodeoxyglucose‐PET as markers of tissue hypoxia. Int J Cancer. 2008;122:2726‐2734. 18351643 10.1002/ijc.23449

[27] Jiang T , Zhou ML , Fan J . Inhibition of GLUT‐1 expression and the PI3K/Akt pathway to enhance the chemosensitivity of laryngeal carcinoma cells in vitro. Onco Targets Ther. 2018;11:7865‐7872. 30464533 10.2147/OTT.S176818 PMC6228052

[28] Xu YY , Wu TT , Zhou SH , et al. Apigenin suppresses GLUT‐1 and p‐AKT expression to enhance the chemosensitivity to cisplatin of laryngeal carcinoma Hep‐2 cells: an in vitro study. Int J Clin Exp Patho. 2014;7:3938‐3947. PMC4129005 25120770

[29] Hamilton DW , Pedersen A , Blanchford H , et al. A comparison of attitudes to laryngeal cancer treatment outcomes: a time trade‐off study. Clin Otolaryngol. 2018;43:117‐1123. 28544805 10.1111/coa.12906

[30] Mayer A , Schneider F , Vaupel P , Sommer C , Schmidberger H . Differential expression of HIF‐1 in glioblastoma multiforme and anaplastic astrocytoma. Int J Oncol. 2012;41:1260‐1270. 22825389 10.3892/ijo.2012.1555 PMC3583842

[31] Swartz JE , Pothen AJ , van Kempen PMW , et al. Poor prognosis in human papillomavirus‐positive oropharyngeal squamous cell carcinomas that overexpress hypoxia inducible factor‐1 alpha. Head Neck. 2016;38:1338‐1346. 27027530 10.1002/hed.24445

[32] Dai LB , Yu Q , Zhou SH , et al. Effect of combination of curcumin and GLUT‐1 AS‐ODN on radiosensitivity of laryngeal carcinoma through regulating autophagy. Head Neck. 2020;42:2287‐2297. 32314842 10.1002/hed.26180

[33] Dai LB , Zhong JT , Shen LF , et al. Radiosensitizing effects of curcumin alone or combined with GLUT1 siRNA on laryngeal carcinoma cells through AMPK pathway‐induced autophagy. J Cell Mol Med. 25(13):6018‐6031. 10.1111/jcmm.16450 33955148

[34] Zhu GC , Peng FS , Gong W , et al. Hypoxia promotes migration/invasion and glycolysis in head and neck squamous cell carcinoma via an HIF‐1 alpha‐MTDH loop. Oncol Rep. 2017;38:2893‐2900. 28901527 10.3892/or.2017.5949

[35] Leung E , Cairns RA , Chaudary N , et al. Metabolic targeting of HIF‐dependent glycolysis reduces lactate, increases oxygen consumption and enhances response to high‐dose single‐fraction radiotherapy in hypoxic solid tumors. BMC Cancer. 2017;17:418. 28619042 10.1186/s12885-017-3402-6 PMC5473006

[36] Zornhagen KW , Hansen AE , Oxboel J , et al. Micro regional heterogeneity of Cu‐64‐ATSM and F‐18‐FDG uptake in canine soft tissue sarcomas: relation to cell proliferation, hypoxia and glycolysis. PLoS One. 2015;10:e0141379. 26501874 10.1371/journal.pone.0141379 PMC4621038

[37] Wang H , Zhao L , Zhu LT , et al. Wogonin reverses hypoxia resistance of human colon cancer HCT116 cells via downregulation of HIF‐1 alpha and glycolysis, by inhibiting PI3K/Akt signaling pathway. Mol Carcinogen. 2014;53:E107‐E118. 10.1002/mc.22052 23761018

[38] Xie Y , Shi X , Sheng K , et al. PI3K/Akt signaling transduction pathway, erythropoiesis and glycolysis in hypoxia (Review). Mol Med Rep. 2019;19:783‐791. 30535469 10.3892/mmr.2018.9713 PMC6323245

[39] Tamburini J , Chapuis N , Bardet V , et al. Mammalian target of rapamycin (mTOR) inhibition activates phosphatidylinositol 3‐kinase/Akt by up‐regulating insulin‐like growth factor‐1 receptor signaling in acute myeloid leukemia: rationale for therapeutic inhibition of both pathways. Blood. 2008;111:379‐382. 17878402 10.1182/blood-2007-03-080796

[40] Chen G , Yuan C , Duan F , et al. IGF1/MAPK/ERK signaling pathway‐mediated programming alterations of adrenalcortex cell proliferation by prenatal caffeine exposure in male offspring rats. Toxicol Appl Pharmacol. 2018;341:64‐76. 29343424 10.1016/j.taap.2018.01.008

[41] Lu J , Tan M , Cai Q . The Warburg effect in tumor progression: Mitochondrial oxidative metabolism as an anti‐metastasis mechanism. Cancer Lett. 2015;356:156‐164. 24732809 10.1016/j.canlet.2014.04.001 PMC4195816

[42] Markowska J , Grabowski JP , Tomaszewska K ,. et al. Significance of hypoxia in uterine cervical cancer. Multicentre study. Eur J Gynaecol Oncol. 2007;28:386‐388. 17966218

[43] Basavaraju AM , Shivanna N , Yadavalli C , Garlapati PK , Raghavan AK . Ameliorative effect of ananas comosus on cobalt chloride‐induced hypoxia in Caco2 cells via HIF‐1alpha, GLUT 1, VEGF, ANG and FGF. Biol Trace Elem Res. 2021;199:1345‐1355. 32654099 10.1007/s12011-020-02278-6

