# Supplementary figures and images for: Effect of Glut‐1 and HIF‐1α double knockout by CRISPR/CAS9 on radiosensitivity in laryngeal carcinoma via the PI3K/Akt/mTOR pathway
Source: J Cell Mol Med. 2022 Apr 12;26(10):2881–94. doi: 10.1111/jcmm.17303 (PMC9907005; doi:10.1111/jcmm.17303)

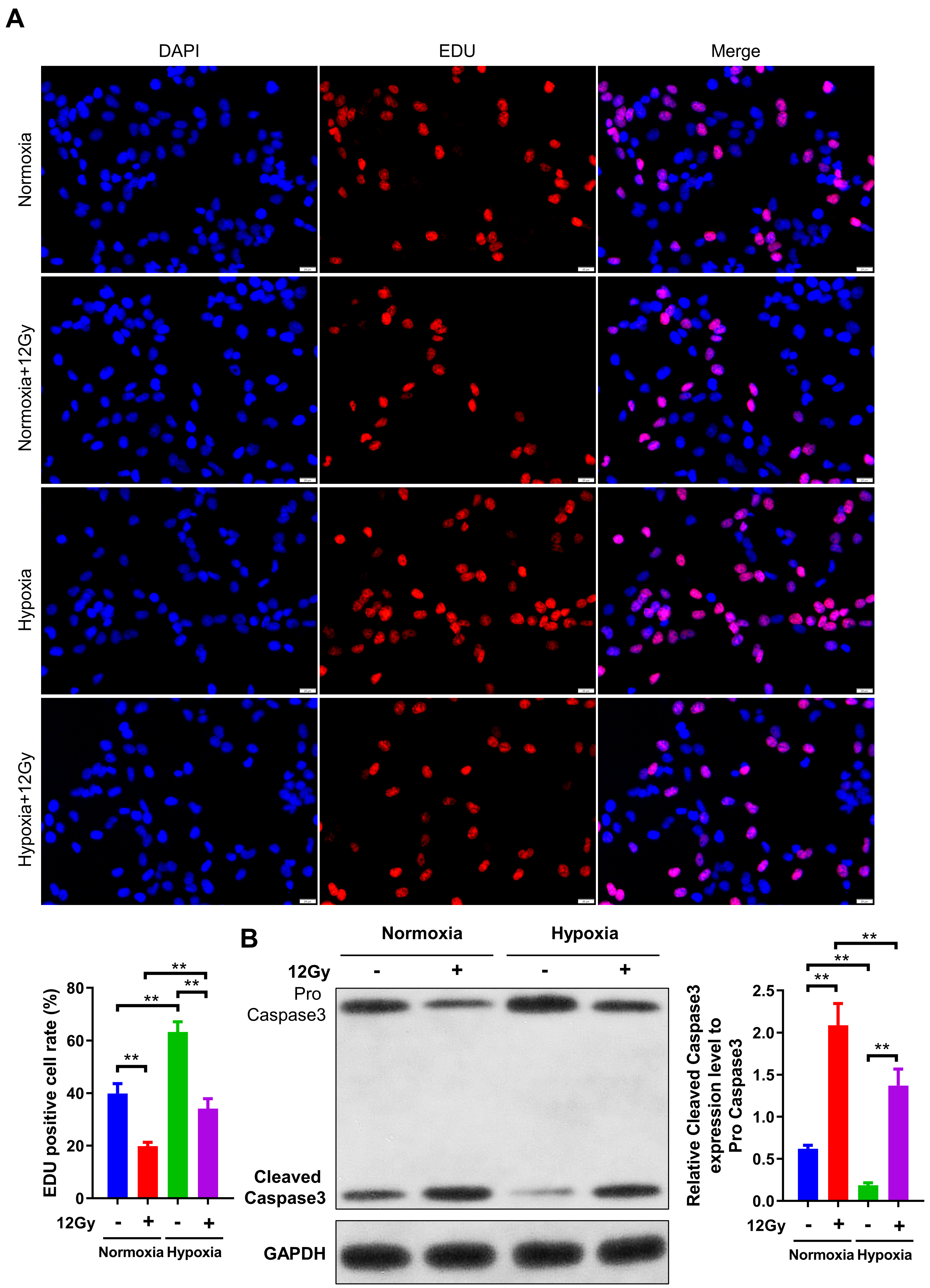

Supplement: Supplementary file 1 — Fig S1 [file JCMM-26-2881-s005.tif]

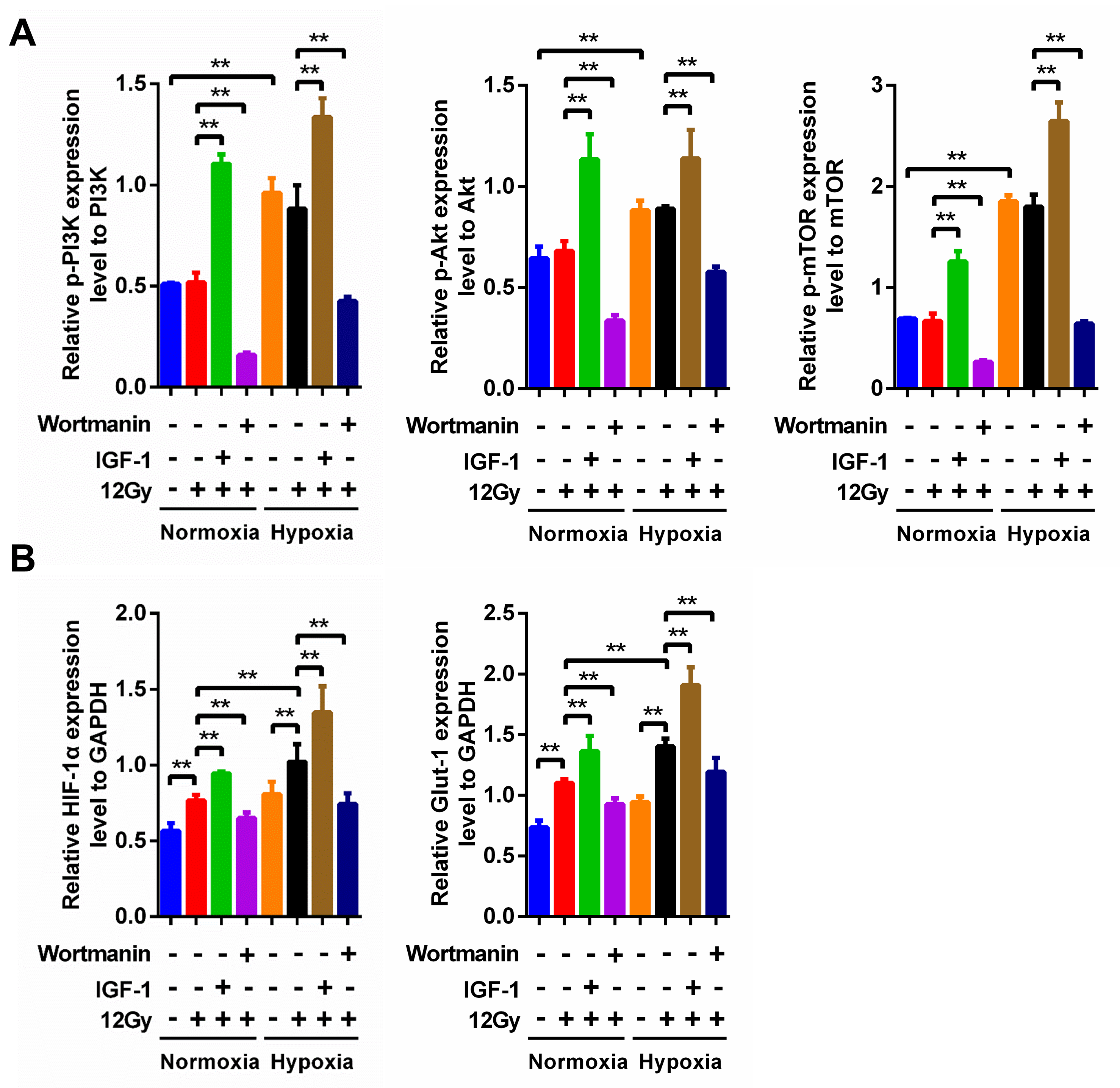

Supplement: Supplementary file 2 — Fig S2 [file JCMM-26-2881-s006.tif]

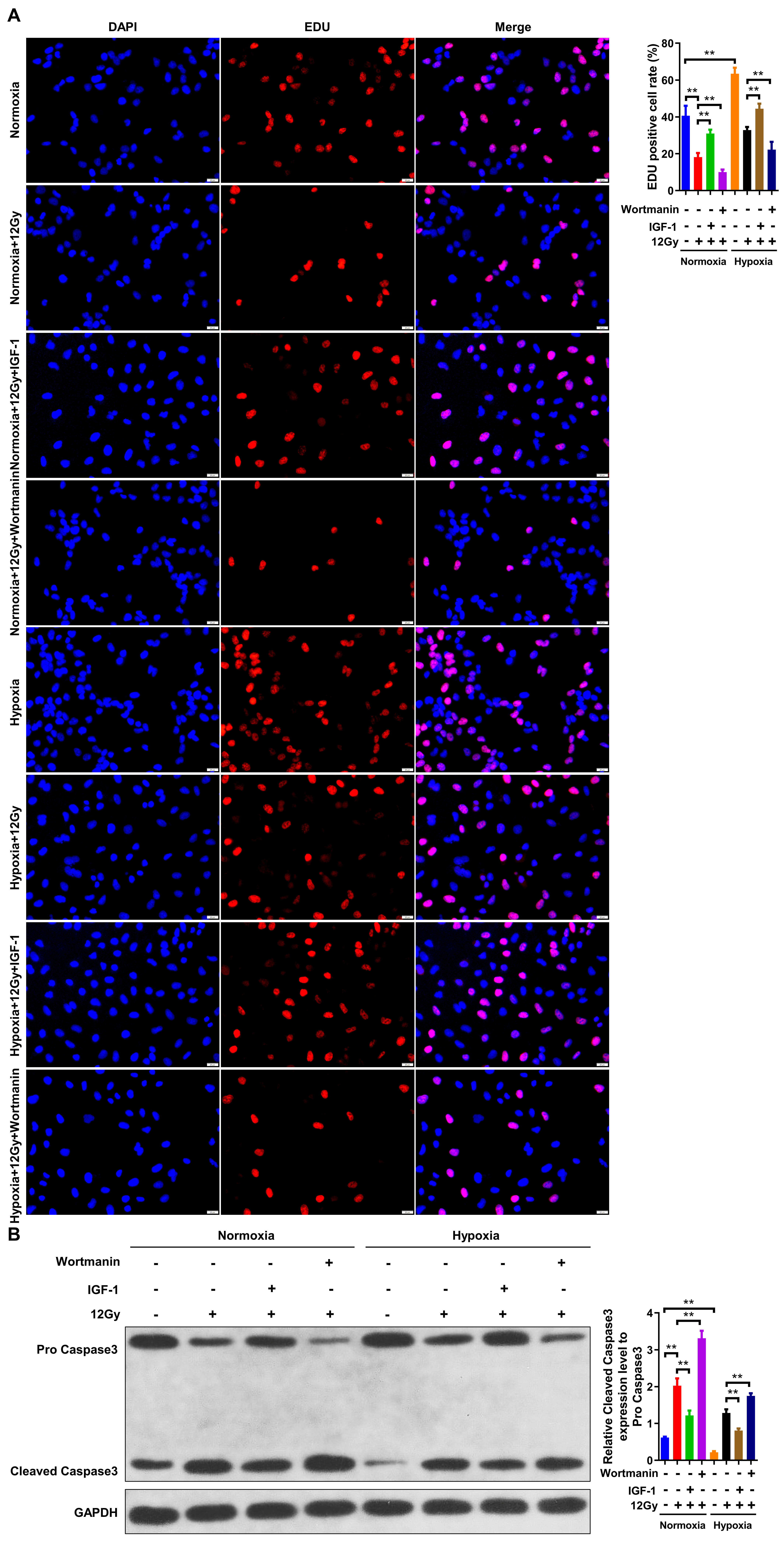

Supplement: Supplementary file 3 — Fig S3 [file JCMM-26-2881-s004.tif]

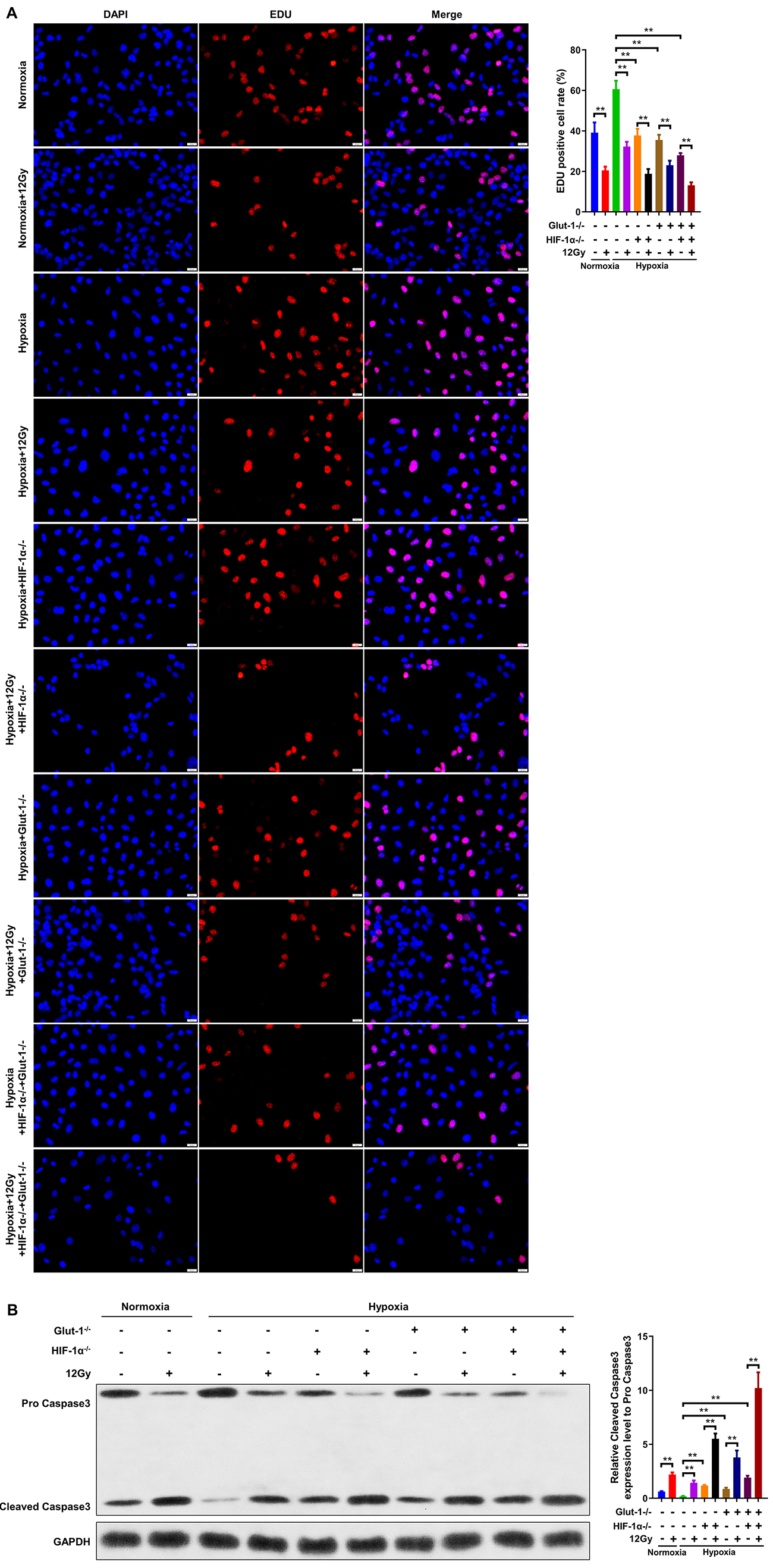

Supplement: Supplementary file 4 — Fig S4 [file JCMM-26-2881-s002.tif]

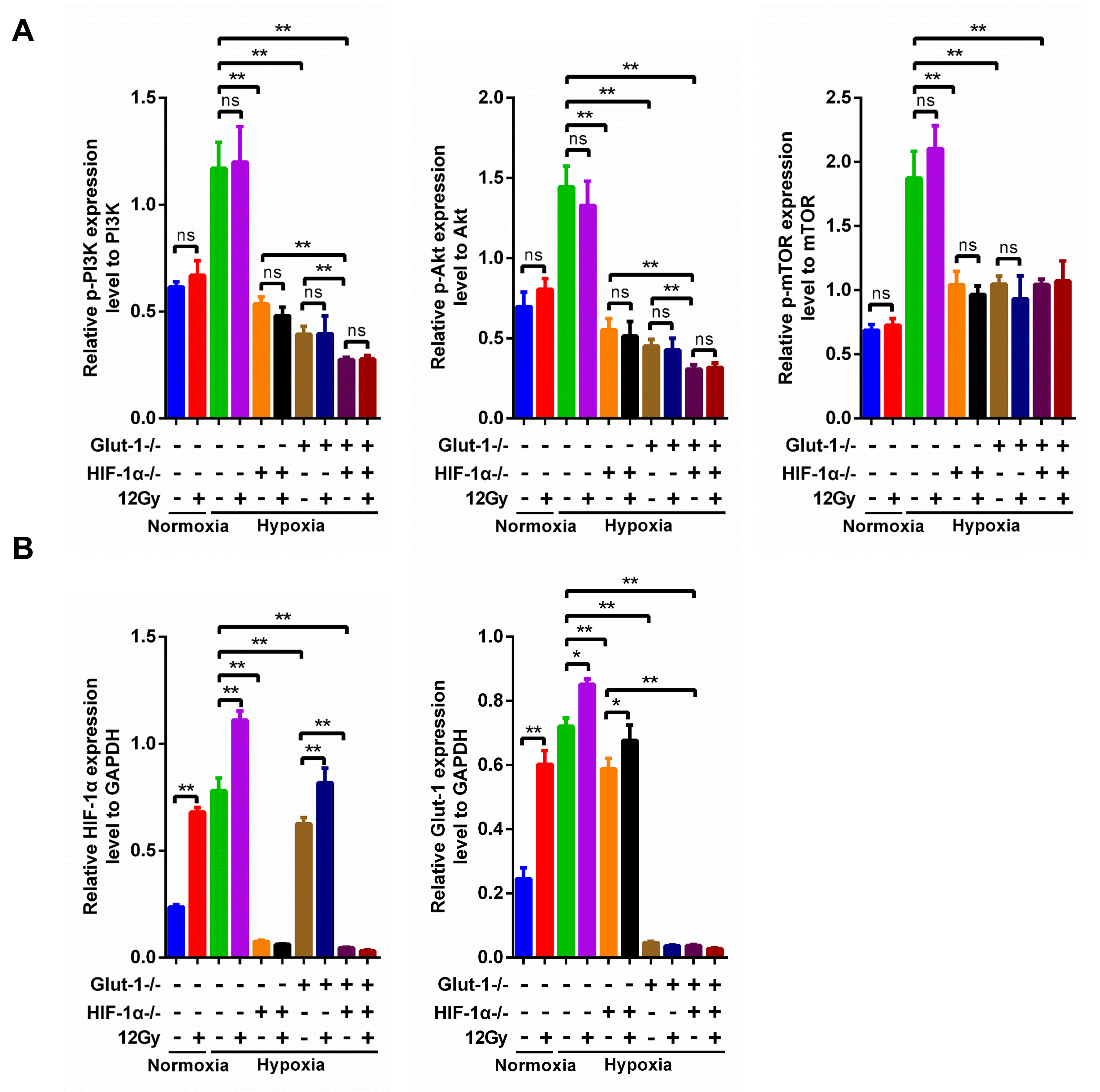

Supplement: Supplementary file 5 — Fig S5 [file JCMM-26-2881-s001.tif]

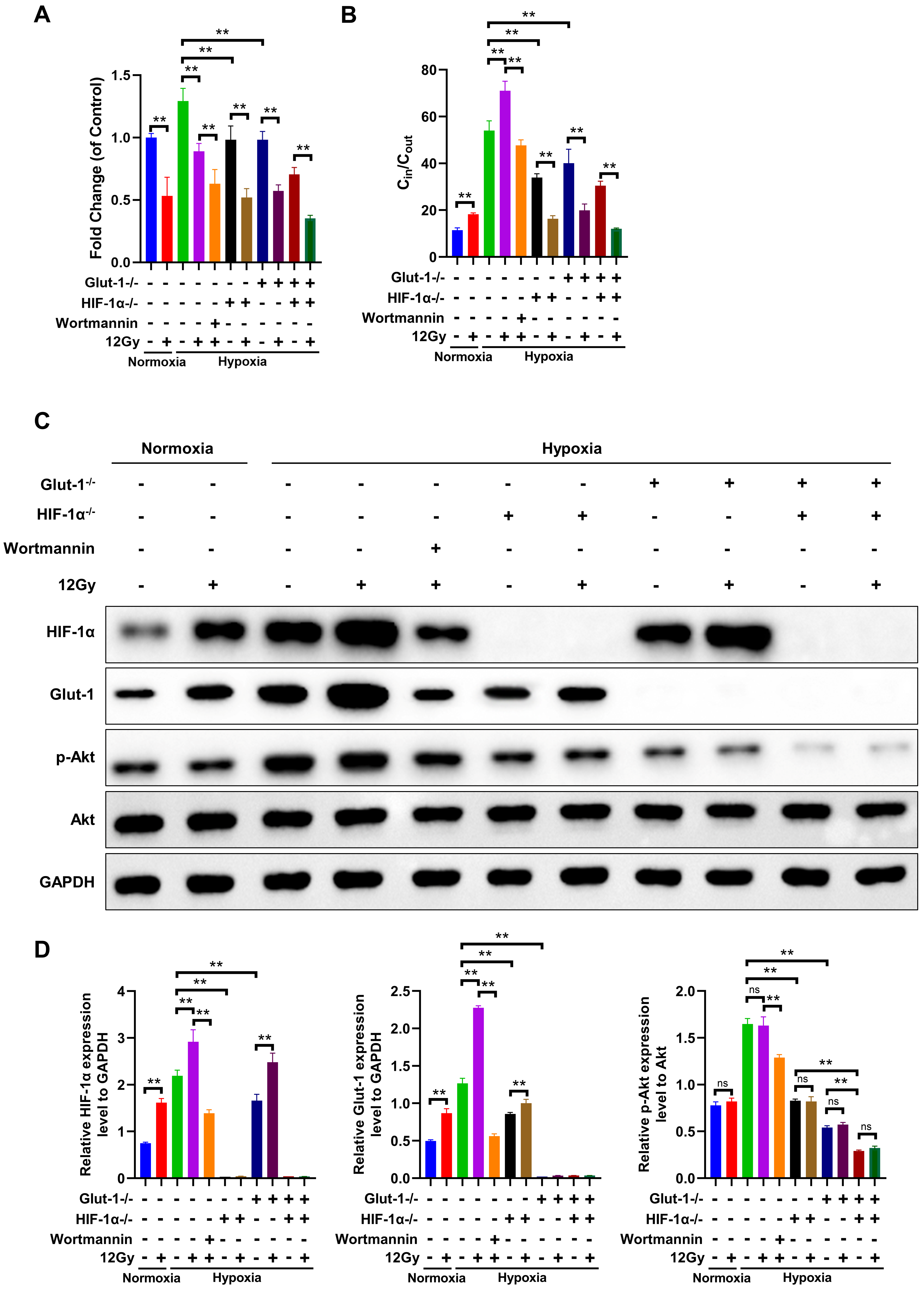

Supplement: Supplementary file 6 — Fig S6 [file JCMM-26-2881-s003.tif]

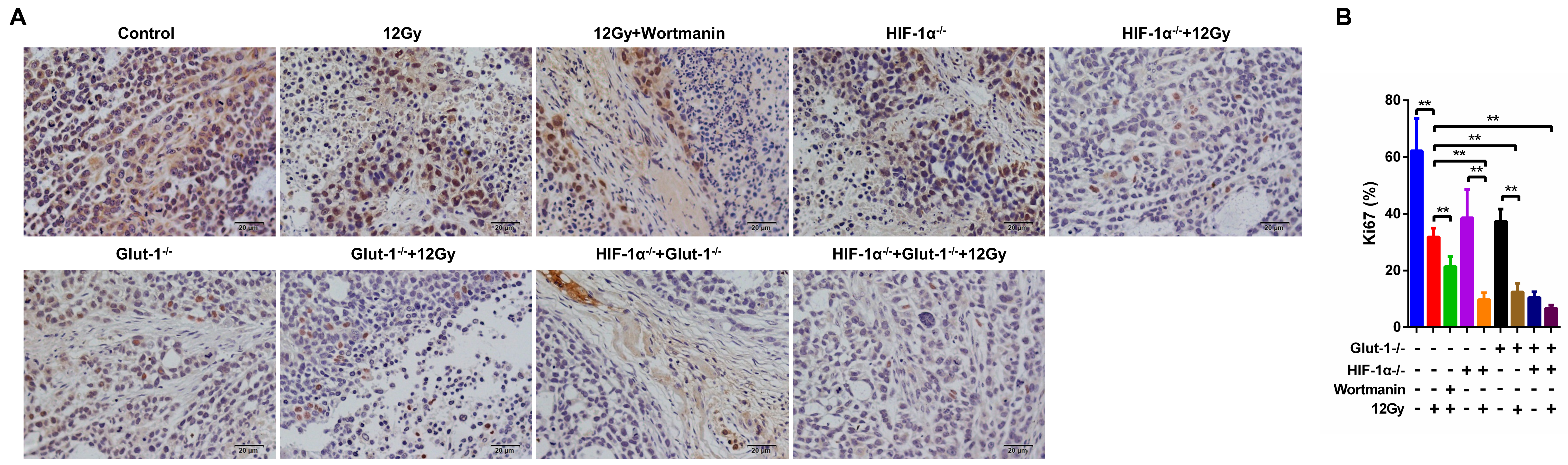

Supplement: Supplementary file 7 — Fig S7 [file JCMM-26-2881-s007.tif]
